# Mycophenolate Mofetil and New-Onset Systemic Lupus Erythematosus: A Randomized Clinical Trial

**DOI:** 10.1001/jamanetworkopen.2024.32131

**Published:** 2024-09-16

**Authors:** Yijun You, Zhuochao Zhou, Fan Wang, Jian Li, Honglei Liu, Xiaobing Cheng, Yutong Su, Xiaowei Chen, Hui Zheng, Yue Sun, Hui Shi, Qiongyi Hu, Jing Xu, Jialin Teng, Chengde Yang, Junna Ye

**Affiliations:** 1 Department of Rheumatology and Immunology, Ruijin Hospital, Shanghai Jiao Tong University School of Medicine, Shanghai, China; 2 Clinical Research Center, Ruijin Hospital, Shanghai Jiao Tong University School of Medicine, Shanghai, China; 3 Department of Rheumatology and Immunology, First Affiliated Hospital of Wenzhou Medical University, Zhejiang, China; 4 Department of Rheumatology and Immunology, Second Affiliated Hospital of Shandong First Medical University, Shandong, China; 5 Department of Nephrology, Ruijin Hospital, Shanghai Jiao Tong University School of Medicine, Shanghai, China

## Abstract

**Importance:**

Anti–double-stranded DNA (dsDNA) antibody has been reported to have a close relationship with systemic lupus erythematosus (SLE) flares and participates in the pathogenesis of lupus nephritis (LN) as well as causing damage to other organs. However, whether early use of mycophenolate mofetil (MMF) could prevent SLE flares is not clear.

**Objective:**

To assess the efficacy and safety of MMF plus prednisone and hydroxychloroquine sulfate compared with prednisone and hydroxychloroquine sulfate alone in patients with SLE.

**Design, Setting, and Participants:**

This investigator-initiated, multicenter, observer-blinded randomized clinical trial enrolled 130 participants aged 18 to 65 years and was conducted in 3 hospitals across China. Treatment-naive patients with newly diagnosed SLE, a high titer of anti-dsDNA antibody, and no major organ involvement were included. The study was started September 1, 2018, and the follow-up was completed September 30, 2021. Data were analyzed from December 1, 2021, to March 31, 2022.

**Interventions:**

Patients were randomized 1:1 to receive oral prednisone (0.5 mg/kg/d) and hydroxychloroquine sulfate (5 mg/kg/d) (control group) or prednisone (0.5 mg/kg/d) and hydroxychloroquine sulfate (5 mg/kg/d) plus MMF (500 mg twice daily) (MMF group) for 96 weeks.

**Main Outcomes and Measures:**

The primary outcome was the proportion of patients presenting with flares according to the Safety of Estrogens in Lupus Erythematosus National Assessment–Systemic Lupus Erythematosus Disease Activity Index (SELENA-SLEDAI) Flare Index. The secondary outcomes included the proportion with lupus low disease activity state at week 96, 36-Item Short Form Health Survey scores before and after treatment, proportion of adverse events (AEs), and changes in SLEDAI-2000 scores and prednisone doses.

**Results:**

Among 130 randomized patients (mean [SD] age, 34.5 [12.5] years; 112 [86.2%] women), 119 (91.5%) completed the follow-up. The risk of severe flare was significantly lower in the MMF group (7 of 65 [10.8%]) vs the control group (18 of 65 [27.7%]) (relative risk [RR], 0.39 [95% CI, 0.17-0.87]; *P* = .01). Additionally, 1 of 65 patients in the MMF group (1.5%) and 9 of 65 in the control group (13.8%) manifested LN (RR, 0.11 [95% CI, 0.01-0.85]; *P* = .008). Most common serious study drug–related AEs were infections (20 of 65 [30.8%] in the control group and 22 of 65 [33.8%] in the MMF group).

**Conclusions and Relevance:**

The findings of this randomized clinical trial suggest that MMF may reduce the rate of severe flare and lower the incidence of LN in patients with new-onset SLE and a high titer of anti-dsDNA antibody without major organ involvement.

**Trial Registration:**

Chinese Clinical Trial Registry: ChiCTR1800017540

## Key Points

**Question:**

What are the efficacy and safety of mycophenolate mofetil (MMF) in patients with new-onset systemic lupus erythematosus (SLE), with high titers of anti–double-stranded DNA antibody, and without major organ involvement?

**Findings:**

This randomized clinical trial of 130 participants found that MMF in addition to oral prednisone and hydroxychloroquine sulfate decreased severe flares and the proportion of patients with kidney involvement compared with patients who received only prednisone and hydroxychloroquine. Furthermore, MMF did not increase incidence of adverse events.

**Meaning:**

These findings suggest that early application of MMF may decrease the rate of severe flare and lower the incidence of lupus nephritis in patients with new-onset SLE, with a high titer of anti–double-stranded DNA antibody, and without major organ involvement.

## Teaser

This randomized clinical trial investigates whether early application of mycophenolate mofetil improves long-term outcomes in patients with newly diagnosed systemic lupus erythematosus with a high titer of anti–double-stranded DNA antibody and without major organ involvement.

## Introduction

Systemic lupus erythematosus (SLE) is a disease characterized by an elevation of large amounts of autoantibodies, including the anti–double-stranded DNA (dsDNA) antibody. The anti-dsDNA antibody could cause organ involvement, including kidney, joint, skin, and so on.^1^ In particular, it contributes to the pathogenesis of lupus nephritis (LN) by binding to antigens in kidney cells or the extracellular matrix, then triggering inflammatory activation and fibrotic processes.^2^ Additionally, kidney involvement has been reported to be more frequent in patients with persistent positivity for anti-dsDNA than in patients with initial positivity and subsequent negative findings for anti-dsDNA or persistent negative findings for anti-dsDNA antibody during the disease course.^3^

Three types of SLE disease activity have been described: relapsing remitting, chronic activity, and long quiescence.^4^ The relapsing-remitting type is the most common SLE disease type. The level of anti-dsDNA antibody fluctuates with disease activity in patients with SLE.^5^ Relapses are preceded by an increase in anti-dsDNA antibody level. In other words, an elevation of anti-dsDNA antibody level may occur before SLE flares even in patients with persistent positivity for anti-dsDNA.^6,7^ One study reported that the prevalence of disease flares ranged from 13.0% to 15.7%.^8^ Therefore, studies are urgently needed to investigate the early prevention of flares in patients with SLE.

Treatment of SLE consists of the use of hydroxychloroquine sulfate, prednisone, and disease-modifying antirheumatic drugs. Mycophenolate mofetil (MMF) is an important disease-modifying antirheumatic drug used in SLE treatment. MMF, a prodrug of mycophenolic acid (MPA), can inhibit inosine monophosphate dehydrogenase, which is responsible for de novo synthesis of guanosine nucleotides.^9^ In addition, MPA can regulate dendritic cell subsets to interrupt the harmful cascade of autoimmune disorders.^10^ Currently, MMF is widely used for induction and maintenance treatment in LN.^11,12^ MMF has been reported to have a role in the treatment of patients with extrarenal involvement.^13,14^ In a multicenter, 24-month randomized clinical trial,^15^ MMF was superior to azathioprine in treating SLE and preventing further relapses. Therefore, this study sought to investigate whether early application of MMF in patients with newly diagnosed SLE, with a high titer of anti-dsDNA antibody, and without major organ involvement could improve long-term prognosis.

## Methods

This report follows the Consolidated Standards of Reporting Trials (CONSORT) reporting guideline for randomized clinical trials. The study protocol and statistical analysis plan are provided in Supplement 1.

### Study Design

This investigator-initiated, multicenter, observer-blinded randomized clinical trial was conducted at the Department of Rheumatology and Immunology in 3 hospitals across China. This study was conducted in accordance with the ethical principles of the Declaration of Helsinki^16^ and the International Conference on Harmonization–Good Clinical Practice guidelines.^17^ The study was approved by the Institutional Research Ethics Committee of Ruijin Hospital and locally authorized ethics committees of the other clinical centers. All patients provided written informed consent. An independent data and safety monitoring board provided oversight. The study was started September 1, 2018, and follow-up was completed September 30, 2021.

### Setting and Participants

In this trial, 205 patients with new-onset SLE were screened. Thereafter, 130 eligible participants aged 18 to 65 years met the inclusion criteria and fulfilled the American College of Rheumatology 2019 classified criteria or its 2017 version.^18^ The diagnosis of SLE was confirmed by 2 senior rheumatologists. During enrollment, patients were required to (1) have received no prior SLE treatment; (2) have a positive antinuclear antibody (HEp-2 titer ≥1:80) finding; (3) have anti-dsDNA antibody positivity (fulfilled both anti-dsDNA with enzyme-linked immunosorbent assay [ELISA] ≥300 IU/mL and *Crithidia luciliae* immunofluorescence test [CLIFT] ≥1:10); and (4) not have major organ involvement (brain, heart, liver, kidney, lung, muscle, and gastrointestinal tract). However, patients with rash, arthritis, alopecia, oral ulcer, and mild hematologic system involvement (white blood cell count, >1500 and <4000 μL [to convert to ×10^9^/L, multiply by 0.001]; hemoglobin level >9.0 and <12.0 g/dL [to convert to g/L, multiply by 10.0]; and platelet count, >60 and <100 × 10^3^/μL [to convert to ×10^9^/L, multiply by 1.0]) (Supplement 1) were not prevented from participation in the study. All serum samples obtained from enrolled patients were sent to the central laboratory of Ruijin Hospital for anti-dsDNA antibody assays (both ELISA and CLIFT).

### Randomization and Masking

Participants were randomly assigned (1:1) using blocks of 4 to receive either MMF (MMF group) or a control treatment (control group). A statistician who was masked to trial allocation generated the randomization sequences by a computer algorithm. Observers were blinded to the allocation process (sealed, opaque envelopes were used to conceal trial allocation). All assessments of participants were evaluated by the observers. Data analysts were not involved in patient assessment and treatment, and they were masked to the group allocation before data analysis.

### Interventions

Patients in the control group were treated with oral hydroxychloroquine sulfate (5 mg/kg/d) and prednisone (0.5 mg/kg/d), whereas the MMF group received hydroxychloroquine sulfate (5 mg/kg/d), prednisone (0.5 mg/kg/d), and MMF (500 mg twice daily). Patients were instructed to take tablets regularly. MMF doses remained unchanged unless a contrary or severe adverse event (AE) occurred. No increased use of antimalarials or immunosuppressants were allowed at any time in this trial if not indicated. The prednisone dose was tapered as follows: the initial dose was maintained for 4 weeks and then tapered by 5.0 mg every 2 weeks. When the dose had been reduced to 20.0 mg/d, it was tapered by 5 mg every month and then gradually to 0.1 to 0.2 mg/kg/d. The tapering of prednisone doses was determined by the physicians based on their assessment of patients. Two physicians formulated a tapering plan for the same time. If there were differences between the 2 physicians’ perspectives, a third experienced senior rheumatologist (C.Y.) made the final decision.

Patients with severe flare were not eligible for the current treatment and needed to be further evaluated by assessors. These patients discontinued the study intervention, but the patient would be followed up during the visiting time. They were treated with increased doses of prednisone (>0.5 mg/kg/d), immunosuppressive therapy, or hospitalization for treatment according to the patient’s condition and the physician’s assessment. However, patients with mild to moderate flares continued to be followed up until the end of week 96, and all flare episodes were recorded. When patients had mild to moderate flares, prednisone (<0.5 mg/kg/d) or nonsteroidal anti-inflammatory drugs could be added. For fever, rash, and arthritis, prednisone could be used at an initial dose of less than 0.5 mg/kg/d; following the assessment of symptoms, the dose could be increased to greater than 0.5 mg/kg/d, if needed. If the patients had any signs of kidney involvement (glomerular hematuria and/or cellular casts, proteinuria of >0.5 g/24 hours, spot urine protein-to-creatine ratio of >500 mg/g, or unexplained decrease in glomerular filtration rate), a kidney biopsy was suggested to confirm the diagnosis of LN. The results of kidney biopsy were confirmed by a senior nephrologist. Lupus nephritis was treated according to the European Renal Association–European Dialysis and Transplant Association guideline.^19^ Other organ involvements were treated following the 2019 European League Against Rheumatism recommendation.^20^

### Outcomes

Patients were assessed on scheduled study visits (baseline and weeks 24, 48, 72, and 96). The primary outcome was the proportion of patients with flares (mild to moderate and severe) during the follow-ups. The Safety of Estrogens in Lupus Erythematosus National Assessment–Systemic Lupus Erythematosus Disease Activity Index (SELENA-SLEDAI) Flare Index was used for assessing disease flares in this trial^4,21^ (eAppendix 1 in Supplement 2). The secondary outcomes included the proportion of lupus low disease activity state (LLDAS) at week 96 (a definition of LLDAS^22^ is provided in eAppendix 2 in Supplement 2), 36-Item Short Form Health Survey (SF-36) scores (range, 0-100; higher scores indicate better mental and physical conditions)^23^ before and after treatment in the 2 groups, proportion of AEs of the 2 groups during follow-ups, changes in SLEDAI-2000 scores (5-9 indicates mild degree of disease activity; 10-14, a moderate degree of disease activity), and changes in prednisone doses. In addition, changes in serological markers, including levels of anti-dsDNA antibody, IgG, and C-reactive protein and erythrocyte sedimentation rate, were assessed. Moreover, the Systemic Lupus International Collaborating Clinics–American College of Rheumatology Damage Index (SDI)^24^ (range, 0-47; higher scores indicate worse organ damage) was compared in the 2 groups.

### Sample Size Calculation

After referring to the flare rate of the placebo group in the Met Lupus trial^25^ and the flare rates from the BLISS (Study of Belimumab in Subjects With Systemic Lupus Erythematosus) 52, BLISS-76, and BLISS–North East Asia phase 3 trials,^26,27^ we considered the flare rates to be 10% in the MMF group and 30% in the control group. The *z* test with unpooled variance was used for sample size calculation. A sample size of 58 patients per group provided the trial with 80% power at a 2-sided α error of .05 to detect a difference between the 2 groups using PASS software, version 11.0 (NCSS Statistical Software). An estimated 10% attrition rate was taken into account, and a sample size of 65 patients per group fulfilled the statistical requirement.

### Statistical Analysis

An intention-to-treat (ITT) approach (including all randomized participants who received ≥1 dose of the intervention treatment) was adopted for the primary and secondary outcomes. The per-protocol population included those who were adherent to trial treatment and excluded patients with important protocol deviations. Participants were analyzed according to the intervention to which they were allocated. The statistical analysis plan was drafted prior to database lock, and it includes a detailed description of the statistical analyses (Supplement 1). The statistical analyses were performed using SPSS Statistics, version 23.0 (IBM Corp); Prism, version 8.0 (GraphPad); and R software, version 4.0.0 (R Project for Statistical Computing). In the descriptive statistics, data were expressed as frequency (percentage) for categorical variables and median (IQR) or mean (SD) for continuous variables. Additionally, a *t* test was used to compare between-group differences in continuous variables after exploring the normality of data distribution using the Shapiro-Wilk test. The Mann-Whitney test was used to compare continuous variables with skewed distributions. The Pearson χ^2^ or Fisher exact test was used to compare between-group differences in categorical variables. Relative risk (RR) was used to compare the proportion of patients with the end point (presenting with flares) between the MMF and control groups. We estimated the severe flare–free survival proportion of patients in the MMF and control groups using the Kaplan-Meier method with a log-rank test. Hazard ratios (HRs) were calculated using the Cox proportional hazards regression model. For the primary outcome assessed at 2 levels (mild to moderate and severe flares), a 2-sided Bonferroni correction was used 2-sided at a significance level of *P* = .025. Other tests were 2-sided, performed at the significance level of *P* = .05. Data were analyzed from December 1, 2021, to March 31, 2022.

## Results

### Baseline Patient Characteristics

**Figure 1.**
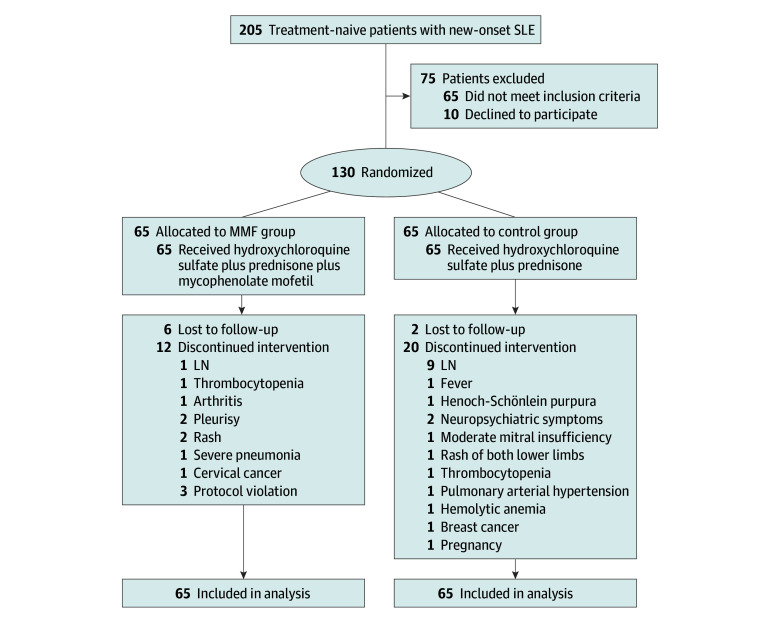
Trial Flow Diagram Patients were followed up for 96 weeks. Flare episodes and adverse events were recorded. LN indicates lupus nephritis; MMF, mycophenolate mofetil; SLE, systemic lupus erythematosus.

Patient disposition is summarized in Figure 1. A total of 205 patients with new-onset SLE who were treatment naive were screened. Seventy-five patients were excluded (65 patients did not meet inclusion criteria and 10 patients declined to participate). A total of 130 patients with SLE met the criteria for enrolment. All patients were randomly assigned (1:1) to the MMF group (n = 65) and the control group (n = 65). The baseline characteristics of patients involved in this study are shown in Table 1. Patients were predominantly female (112 [86.2%] compared with 18 [13.8%] male) with a mean [SD] age of 34.5 (12.5) years. Median age in both groups was 32 years (IQR, 27-46 years in the control group and 23-44 years in the MMF group). Overall, 119 patients completed the study (63 [96.9%] in the control group and 56 [86.2%] in the MMF group). Twelve patients discontinued the intervention in the MMF group, including 3 due to failure to continue to take MMF, and 6 were lost to follow-up. In the control group, 20 patients discontinued the intervention and 2 were lost to follow-up. In terms of the clinical characteristics, levels of anti-dsDNA antibody (257.4 [238.8] vs 563.8 [287.6]; 294.2 [212] vs 642.4 [328.5]) and IgG (1550 [620] vs 2155 [940.3]; 1325 [275] vs 1910 [668]) and ESR erythrocyte sedimentation rate (14 [15] vs 33 [37.3]; 13 [14] vs 42.5 [42.5]) levels were improved compared with their baseline levels (Week 24 to baseline, median [IQR], control group vs MMF group; all *P* < .05) (eFigure 1 in Supplement 2). However, no significant difference in C-reactive protein level was observed (eFigure 1 in Supplement 2).

**Table 1.** Demographic and Baseline Characteristics of Patients in the Control and MMF Groups

Characteristic	Participants, No. (%)^a^	
	Control (n = 65)	MMF (n = 65)
Age, median (IQR), y	32 (27-46)	32 (23-44)
Sex		
Female	55 (84.6)	57 (87.7)
Male	10 (15.4)	8 (12.3)
Disease duration, median (IQR), mo	3 (1-12)	4 (1-24)
SLEDAI-2000 score, median (IQR)^b^	9 (6-11)	10 (8-14)
System involvement		
Arthritis	36 (55.4)	47 (72.3)
Rash	24 (36.9)	22 (33.8)
Fever	22 (33.8)	19 (29.2)
Alopecia	9 (13.8)	16 (24.6)
Oral ulcer	5 (7.7)	8 (12.3)
Leukopenia	34 (52.3)	25 (38.5)
Thrombocytopenia	17 (26.2)	18 (27.7)
Laboratory results		
Anti-dsDNA antibody, median (IQR), U/mL	563.8 (470.9-758.5)	642.4 (459.3-787.8)
Low C3 complement protein level	31 (47.7)	45 (69.2)
Low C4 complement protein level	42 (64.6)	47 (72.3)
WBC count, median (IQR), cells/μL	3600 (2700- 5500)	4100 (2700-5900)
Hemoglobin level, mean (SD), g/L	117.3 (16.2)	114.9 (19.8)
Platelet count, mean (SD), 10^3^/μL	153.3 (71.5)	174.2 (89.1)
ESR, median (IQR), mm/h	33 (21.0-58.3)	42.5 (20.5-63.0)
CRP level, median (IQR), mg/dL	0.10 (0.02-0.35)	0.12 (0.04-0.56)

Abbreviations: CRP, C-reactive protein; dsDNA, double-stranded DNA; ESR, erythrocyte sedimentation rate; MMF, mycophenolate mofetil; SLEDAI, Systemic Lupus Erythematosus Disease Activity Index; WBC, white blood cell.

SI conversion factors: To convert CRP to mg/L, multiply by 10; platelets to ×10^9^/L, multiply by 1.0; and WBC to ×10^9^/L, multiply by 0.001.

^a^ Unless otherwise indicated. Percentages have been rounded and may not total 100.

^b^ Scores of 5 to 9 indicate a mild degree of disease activity; 10 to 14, moderate degree of disease activity.

### Primary Outcome

**Figure 2.**
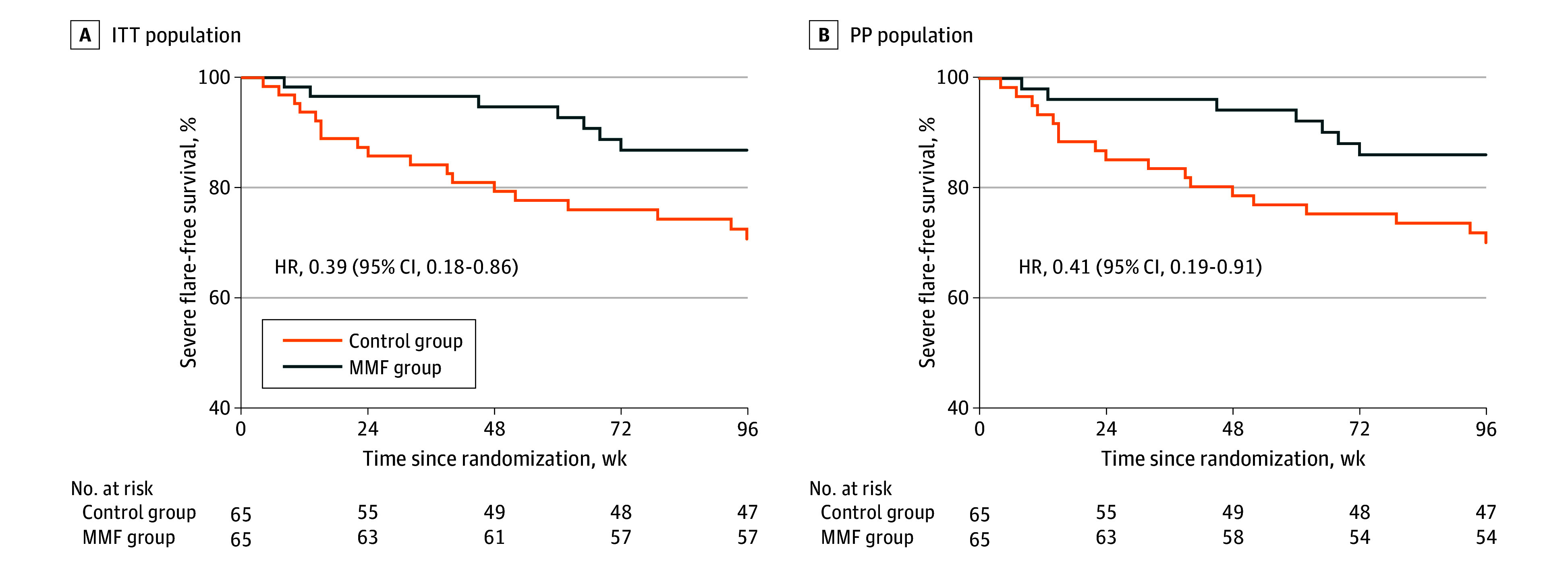
Kaplan-Meier Analysis of Severe Flare–Free Survival HR indicates hazard ratio; ITT, intention-to-treat; MMF, mycophenolate mofetil; and PP, per protocol.

During follow-up, the control group manifested more total flares than the MMF group in the ITT population (RR, 0.68 [95% CI, 0.49-0.96]; *P* = .02) (Table 2). The estimated risk reduction of severe flares in the MMF group was 7 (10.8%) vs 18 (27.7%) in the control group (RR, 0.39 [95% CI, 0.17-0.87]; *P* = .01, Bonferroni correction). Furthermore, in the control group, 9 patients (13.8%) presented with LN compared with 1 (1.5%) in the MMF group (RR, 0.11 [95% CI, 0.01-0.85]; *P* = .008). Among these 10 patients, 1 patient in the control group declined the kidney biopsy, and the remaining 9 patients underwent kidney biopsy and were confirmed to have LN (eTable 1 in Supplement 2). A Kaplan-Meier analysis demonstrated that the severe flare–free survival of patients in the MMF group was higher than that of patients in the control group (HR, 0.39 [95% CI, 0.18-0.86]) (Figure 2). However, no significant difference in the risk of mild to moderate flares was observed between the 2 groups (RR, 0.96 [95% CI, 0.62-1.49]; *P* = .90). Together, these results demonstrated that patients in the MMF group had less severe flares and less kidney involvement than patients in the control group.

**Table 2.** Outcomes of the Control and MMF Groups After 96-Week Follow-Up According to the SFI

Outcome	Participant group, No. (%)		RR (95% CI)	*P* value
	Control (n = 65)	MMF (n = 65)		
**Primary outcome**				
All flares	41 (63.1)	28 (43.1)	0.68 (0.49-0.96)	.02
Mild to moderate flares	25 (38.5)	24 (36.9)	0.96 (0.62-1.49)	.90^a^
Severe flares	18 (27.7)	7 (10.8)	0.39 (0.17-0.87)	.01^a^
**Severe flares**				
New or worsening symptoms^b^				
Lupus nephritis	9 (13.8)	1 (1.5)	0.11 (0.01-0.85)	.008
Neuropsychiatric symptoms	2 (3.1)	0	NA	.20
Thrombocytopenia (<60 × 10^3^/μL)	1 (1.5)	1 (1.5)	NA	>.99
Hemolytic anemia	1 (1.5)	0	NA	.30
Change in SELENA-SLEDAI score >12				
Pulmonary arterial hypertension	1 (1.5)	0	NA	.30
Henoch-Schönlein purpura	1 (1.5)	0	NA	.30
Moderate mitral insufficiency	1 (1.5)	0	NA	.30
Pleurisy	0	2 (3.1)	NA	.20
Increased use of prednisone^c^				
Fever (dose >0.5 mg/kg/d)	1 (1.5)	0	NA	.30
Rash (dose >0.5 mg/kg/d)	1 (1.5)	2 (3.1)	NA	.60
Arthritis (dose >0.5 mg/kg/d)	0	1 (1.5)	NA	.30
**Mild to moderate flares**				
New or worsening symptoms^d^				
Arthritis	20 (30.8)	17 (26.2)	0.85 (0.49-1.47)	.60
Rash	1 (1.5)	3 (4.6)	3.00 (0.32-28.09)	.30
Oral ulcer	0	1 (1.5)	NA	.30
Fever	1 (1.5)	0	NA	.30
Variation of SELENA-SLEDAI score ≥3 (but <12)				
Leukopenia (WBC count, 1540/μL)	2 (3.1)	2 (3.1)	NA	>.99
Thrombocytopenia (platelet count, 60-100 × 10^3^/μL)	1 (1.5)	1 (1.5)	NA	>.99
Increase in the dose of prednisone but <0.5 mg/kg/d				
Arthritis (dose <0.5 mg/kg/d)	20 (30.8)	17 (26.2)	0.85 (0.49-1.47)	.60
Fever	1 (1.5)	0	NA	.30
Increase of nonsteroidal anti-inflammatory drugs or hydroxychloroquine sulfate				
Arthritis	3 (4.6)	2 (3.1)	NA	.70

Abbreviations: MMF, mycophenolate mofetil; NA, not applicable; RR, relative risk; SELENA-SLEDAI, Safety of Estrogens in Lupus Erythematosus National Assessment–Systemic Lupus Erythematosus Disease Activity Index; WBC, white blood cell.

SI conversion factors: To convert platelets to ×10^9^/L, multiply by 1.0; WBC to ×10^9^/L, multiply by 0.001.

^a^ Bonferroni correction.

^b^ Includes new or worsening central nervous system involvement, vasculitis, nephritis, myositis, thrombocytopenia (platelet count <60 × 10^3^/μL), or hemolytic anemia (hemoglobin level <7.0 g/dL or decrease >3.0 g/dL [to convert to g/L, multiply by 10.0]).

^c^ Indicates doubled in dosage or more than 0.5 mg/kg/d.

^d^ Include rashes, cutaneous vasculitis, nasopharyngeal ulcers, serositis, arthritis, or fever caused by lupus.

### Secondary Outcomes

At the end of the follow-up, 27 patients (41.5%) in the MMF group achieved LLDAS compared with 23 (35.4%) in the control group (RR, 1.17 [95% CI, 0.76-1.82]; *P* = .50). Moreover, at baseline, the mean (SD) SLEDAI-2000 scores of the control group (9.4 [4.3]) and the MMF group (11.2 [4.6]) suggested mild to moderate disease activity. At week 24, the SLEDAI-2000 scores of both groups decreased to less than 4 and where they continued to week 96 (eFigure 1 and eTable 5 in Supplement 2). At the last visit, mean (SD) SLEDAI-2000 scores in the control and MMF groups were 2.7 (3.3) and 2.6 (3.0), respectively, indicating a stable low disease activity.

The SF-36 score is a commonly used tool for assessing patients’ quality of life. Patients in the MMF group had better SF-36 scores than those in the control group (eFigure 2 and eTable 2 in Supplement 2). In particular, they exhibited a better improvement in physical, bodily pain, and social functioning (score variation >8). The results showed that SF-36 scores improved in both the control and MMF groups after treatment but without any significant difference between them (eTable 2 in Supplement 2).

The SDI was used to assess cumulative damage across multiple organs, regardless of any causes.^24,28,29^ Five patients in the control group met the criteria of SDI compared with 2 patients in the MMF group (RR, 0.40 [95% CI, 0.08-1.99]; *P* = .30) (eTables 3 and 4 in Supplement 2).

### Adverse Events

We investigated the occurrence of AEs in both groups (Table 3). No difference in total AEs was observed between the control (23 [35.4%]) and MMF (30 [46.2%]) groups (RR, 1.30 [95% CI, 0.86-1.99]; *P* = .20). Infections were the most common AEs, present in 22 patients (33.8%) in the MMF group and 20 (30.8%) in the control group (RR, 1.10 [95% CI, 0.67-1.81]; *P* = .70). No significant difference in any AE item was observed between groups.

**Table 3.** Adverse Events in the Control and MMF Groups

Adverse event	Participant group, No. (%)		*P* value
	Control (n = 65)	MMF (n = 65)	
All	23 (35.4)	30 (46.2)	.20
Infection	20 (30.8)	22 (33.8)	.70
Upper respiratory tract	16 (24.6)	19 (29.2)	.60
Pneumonia	0	2 (3.1)	.20
Lower urinary tract	6 (9.2)	2 (3.1)	.10
Herpes zoster virus	2 (3.1)	4 (6.2)	.40
* Candida* species	1 (1.5)	0	.30
Tuberculosis	0	1 (1.5)	.30
Gastrointestinal tract	11 (16.9)	11 (16.9)	>.99
Bone fracture	0	1 (1.5)	.30
Osteonecrosis of the femoral head	0	1 (1.5)	.30
Other events	6 (9.2)	11 (16.9)	.20

Abbreviation: MMF, mycophenolate mofetil.

## Discussion

The clinical manifestations of SLE are heterogeneous and involve 1 or several organs, including the skin, kidney, joint, and hematologic and nervous systems.^30^ Anti-dsDNA antibody has been widely recognized to be involved in the pathogenesis of LN and other organ involvement.^2,5^ Therefore, anti-dsDNA antibody level is an important parameter in SLE. This is the first study, to our knowledge, to explore early application of MMF in treatment-naive patients with new-onset SLE with a high titer of anti-dsDNA antibody. It demonstrated that MMF reduced severe flare risk (10.8% vs 27.7% in the MMF and control groups, respectively); thus, patients might experience less severe flares and achieve favorable long-term outcomes.

MMF is well known for the induction and maintenance treatment of LN.^12,31,32^ In this study, MMF also performed better in reducing LN in the MMF group compared with the control group. Another study showed that after the initial induction phase of LN treatment, maintenance therapy with azathioprine or MMF was usually given for at least 2 to 3 years, with the latter being slightly more efficacious with fewer relapses.^33,34,35^ Additionally, MMF is widely used and safe for treatment of patients with SLE with extrarenal involvement.^13,36,37,38^ Previous research showed that MPA, the active form of MMF, mediates reversible inhibition of B- and T-cell proliferation without causing myelotoxicity.^39^ Mycophenolic acid was found to have antiviral and anti-inflammatory properties.^40,41^ In addition, MPA can also affect fibroblast biology.^42^ Our results also indicated that among patients with a high titer of anti-dsDNA antibody, aside from the standard treatment using hydroxychloroquine and prednisone, early use of low-dose MMF might be beneficial for patients with SLE.

The Asian LN network suggests a MMF dose of 2.0 g/d and indicates a potential reduction to 1.0 to 1.5 g/d when an improvement is observed after induction.^43^ Monitoring of MMF dosing might become even more important in Asian patients for whom high-dose MMF (3.0 g/d) was not well tolerated, leading to higher infection risk and mortality.^44^ Given previous research findings, we chose a relatively small dose of MMF (1.0 g/d) for early treatment in the MMF group.

Despite decreases in severe flare and incidence of LN in the MMF group, however, there was no difference in LLDAS between groups. What we presented was the proportion of LLDAS at week 96. In this study, patients with LN were diagnosed before week 96. After treatment, they could still achieve LLDAS at week 96. Thus, there was no difference in LLDAS. In addition, no significant difference was observed in organ damage between the control and MMF groups. It might indicate that we should follow up these patients for a longer time. Moreover, SDI is a relatively strict parameter, and its criteria involve severe organ damage. For example, patients who were diagnosed with LN in our study still did not meet the standard of SDI kidney damage.

### Limitations

This trial has several limitations. First, a relatively small number of patients were included because of strict enrolment criteria, which required treatment-naive patients with new-onset SLE, a high titer of anti-dsDNA antibody, and no major organ involvement. Second, despite the 96-week follow-up period, a study involving a longer follow-up period is still needed to determine the advantages and disadvantages of early application of MMF. Third, our study was conducted among Asian patients. Therefore, we could not ascertain whether our findings could be generalized to other groups. Further studies in more diverse patient populations should be conducted. Fourth, this study was an open-label, observer-blinded study. A double-blind, placebo-controlled study is required to confirm our findings.

## Conclusions

The preliminary findings from this randomized clinical trial indicated that among treatment-naive patients with new-onset SLE, a high titer of anti-dsDNA antibody, and no organ involvement, early use of low-dose MMF may decrease the risk of severe flare and incidence of LN. Further investigation is warranted to assess the balance between potential benefits and harms.

## Notes

Supplement 1.
Trial Protocol and Statistical Analysis Plan

Supplement 2. **eTable 1.** Histopathological Details on LN Classification in Control Group and MMF Group **eTable 2.** Comparison of SF-36 Scores Before and After Treatment in Control Group and MMF Group **eTable 3.** The Comparison of Organ Damage in Control Group and MMF Group During 96-Week Follow-Up According to SDI for SLE **eTable 4.** The Details of Organ Damage in Control Group and MMF Group During 96-Week Follow-Up According to SDI for SLE **eTable 5.** SLEDAI-2000 and Prednisone Dose in Control Group and MMF Group at Week 96 **eAppendix 1.** List of Safety of Estrogens in Lupus Erythematosus National Assessment-Systemic Lupus Erythematosus Disease Activity Index (SELENA-SLEDAI) Flare Index (SFI) **eAppendix 2.** List of LLDAS Definition **eFigure 1.** Clinical Features of the Control and MMF Groups **eFigure 2.** Pretreatment and Posttreatment SF-36 Scores in Control Group and MMF Group

Supplement 3.
Data Sharing Statement
