# Supplementary material for: Mycophenolate Mofetil and New-Onset Systemic Lupus Erythematosus: A Randomized Clinical Trial
Source: JAMA Netw Open. 2024 Sep 16;7(9):e2432131. doi: 10.1001/jamanetworkopen.2024.32131 (PMC11406395; doi:10.1001/jamanetworkopen.2024.32131)
Supplement: Supplement 1. — Trial Protocol and Statistical Analysis Plan [file jamanetwopen-e2432131-s001.pdf]

Mycophenolate mofetil and new-onset systemic lupus erythematosus with high titer of anti-dsDNA antibody and without major organ involvement: A Randomized Clinical Trial

Protocol

Version 2.0

Principal Investigator:

Junna Ye, MD & PhD

Department of Rheumatology and Immunology, Ruijin Hospital, Shanghai Jiao Tong University School of Medicine

No. 197 Ruijin Second Road, Huangpu District, Shanghai 200025, China.

Tel. : +86 21 64370045 ;

Fax : +86 21 34186000 ;

E-mail : yjn0912@qq.com

Amendment records

Date of revision	Version	Main modification
2020.08.09	2.0	4 hospitals was not in the list because no patients was included<br><br>Ajusted the sample size to 65 per group

TABLE OF CONTENTS

Amendment records 2
TABLE OF CONTENTS 3
STUDY GLOSSARY 5
SUMMARY 6
Application Form for Participation in Research 9
I. Letter of Commitment 9
II. Brief list 10
III.Detailed description of research activities 11
1. Research abstract 11
1.1 Abstract 11
1.2 Workflow 14
First survey 14
Second survey 14
Third survey 14
Fourth survey 14
Fifth survey 14
2. Research Background 15
2.1 Research Significance 15
2.2 Research Background 15
2.3 Expected Outcomes of the Research 17
2.4 Risk/Benefit Assessment 17
2.4.1 Potential risks 17
2.4.2 Known potential benefit 17
2.4.3 Potential Risk/Benefit Assessment 17
3. Principal Investigator Information 18
3.1 Principal investigator name, qualifications, contact information, brief introduction 18
3.2 Main participants 18
4. Research objectives 19
5. Research design 20
5.1 General design 20
5.2 Define research endpoints 20
5.3 Determining sample size 21
6. Research object 21
6.1 Inclusion criteria 21
6.2 Exclusion criteria 22
6.3 Things to pay attention to in life 22
6.4 Recruitment of patients 22
6.5 Methods of subject assignment 23
7. Research interventions 23
7.1 Research interventions 23
7.1.1 Description of Intervention 23
7.1.2 Dosage and Administration 24
7.1.3 Establishment, preservation, and unblinding methods of trial drug codes and methods of unblinding in emergency situations 24
7.1.4 Items and times of clinical and laboratory examinations to be carried out 24
7.2 Preparation/handling/storage/responsibility 24
7.2.1 Responsibility 24

7.2.2 Composition, Appearance, Packaging and Labeling 24
7.2.3 Product Storage and Stability.. 24
7.2.4 Preparation 24
7.3 Measures to reduce bias: randomization and blinding..................................... 25
7.4 Follow-up and compliance... 25
7.5 Research Intervention Commitment 25
7.6 Research plan 25
8. Study Intervention Discontinuation and Study Subject Discontinuation/Withdrawal 26
8.1 Research Intervention Discontinuation.. 26
8.2 Subject discontinuation/withdrawal... 27
8.3 Lost to follow-up.. 27
9. Evaluation of research outcomes 27
9.1 Primary and secondary outcome evaluation 27
9.2 Safety and other evaluations 27
9.3 Adverse events and serious adverse events 27
9.3.1 Adverse Event (AE) Definition 27
9.3.2 Serious Adverse Event (SAE) Definition 28
9.3.3 Adverse event classification 28
9.3.3.1 Incident Severity 28
9.3.3.2 Relevance to Research Interventions 28
9.3.3.3 Expectancy 28
9.3.4 Timing, frequency, follow-up, and regression of adverse event assessments28
9.3.5 Adverse Event Reporting... 29
9.3.6 Serious Adverse Event Reporting... 29
9.3.7 Reporting adverse events to study subjects 29
9.4 Unexpected problem 29
9.4.1 Definition of Unexpected Problems... 29
9.4.2 Reporting of unexpected problems 29
10. Statistical analysis.. 29
10.1 General method.. 29
10.2 Analysis of primary and secondary research endpoints................................. 29
10.3 Security Analysis 30
10.4 Baseline Descriptive Analysis 30
10.5 Subgroup analysis 30
11. Supporting Documents and Notices.. 30
11.1 Informed Consent Process 30
11.2 Research Termination and Closure... 30
11.3 Privacy protection 30
11.4 Collection and use of specimens and data 31
11.5 Quality Control and Quality Assurance... 31
11.6 Data processing and record keeping 32
11.6 Data collection and management.. 32
11.7 Publishing and Data Sharing Agreements 32
11.8 Conflict of Interest Statement... 32
12. Appendix... 32
12.1 Workflow of glucocorticoids decrease process from baseline to week 96..... 32
12.2 SF-36 questionnaire 33
12.2.1 SF-36 questionnaire Chinese version.. 33
12.2.2 SF-36 questionnaire.. 36

STUDY GLOSSARY

ACR	American College of Rheumatology
AE	Adverse event
ALT	Alanine aminotransferase
AST	Aspartate aminotransferase
CLIFT	Crithidia luciliae immunofluorescence test
CRF	Case report form
CRP	C-Reactive Protein
CTCAE	Common Terminology Criteria for Adverse Events
ELISA	Enzyme-linked immunosorbent assay
ERA-EDTA	European Renal Association-European Dialysis and Transplant Association
ESR	Erythrocyte sedimentation rate
EULAR	European League Against Rheumatism
GCP	Good Clinical Practice
GFR	Glomerular filtration rate
HCQ	Hydroxychloroquine
IL-1	Interleukin-1
IL-2	Interleukin-2
IL-6	Interleukin-6
ICH-GCP	International Conference on Harmonization-Good Clinical Practice
LLDAS	Lupus low disease activity state
LN	Lupus nephritis
MHC	Major histocompatibility complex
MMF	Mycophenolate mofetil
SAE	Serious Adverse Event
SD	Standard deviations
SELENA-SLEDAI	Safety of Estrogens in Lupus Erythematosus National Assessment-Systemic Lupus Erythematosus Disease Activity Index
SF-36	Short form-36
SLE	Systemic lupus erythematosus
SLEDAI-2000	SLE Disease Activity Index-2000
TNF-α	Tumor necrosis factor-α
UPCR	Urine protein-to-creatine ratio

SUMMARY

**TITLE:**	A randomized controlled trial for the long-term outcomes of MMF on new onset SLE patients with high titers of anti-dsDNA antibody for reducing main organ involvement
**SPONSOR**	Department of Rheumatology and Immunology, Ruijin Hospital, Shanghai Jiao Tong University School of Medicine
**COORDINATING INVESTIGATOR**	Principal Investigator:<br>Junna Ye, MD & PhD<br>Department of Rheumatology and Immunology, Ruijin Hospital, Shanghai Jiao Tong University School of Medicine<br>No. 197 Ruijin Second Road, Huangpu District, Shanghai 200025, China.<br>Tel. : +86 21 64370045 ;<br>Fax : +86 21 34186000 ;<br>mail : yjn0912@qq.com
**PROTOCOL VERSION**	Version 2.0
**BACKGROUND AND SCIENTIFIC RATIONALE**	Systemic lupus erythematosus (SLE) is an autoimmune disease that can affect multiple organs in the body. The abnormality of the immune system is manifested by the existence of a large number of circulating self-antigens in the body, resulting in the production of autoantibodies against various self-antigens and causing immune damage to organs. Among them, anti-dsDNA antibodies are the most important autoantibodies, and high titers of anti-dsDNA antibodies are closely related to the pathogenesis of lupus nephritis, and can cause damage to other multiple organs, such as liver, heart, and lungs. Studies have shown that the incidence of SLE in the Asian population is about 8.4/100,000 people/year, and severe cases can even be life-threatening, bringing a significant economic burden to people's lives and economy. As a commonly used immunosuppressant, mycophenolate mofetil (MMF) has been widely used as a drug for induction and maintenance of lupus nephritis, and also plays an important role in the treatment of other extra-renal organ damage.<br>Therefore, we propose the scientific hypothesis that whether early application of MMF to newly diagnosed SLE patients with high titer of anti-dsDNA antibody and without major organ involvement could improve the long-term prognosis.
**PRIMARY OUTCOME**	The proportion of SLE patients having flares (mild-to-moderate flare and severe flare) according to SELENA-SLEDAI Flare Index.
**SECONDARY OUTCOMES**	1) The proportion of lupus low disease activity state (LLDAS) at week 96;<br>2) Short form-36 (SF-36) score before and after treatment;<br>3) The proportion of adverse events in the two groups during follow-ups;<br>4) Changes in SLEDAI-2000 score;<br>5) Changes in prednisone dose.
**STUDY DESIGN**	Multicenter randomized clinical trial

In this study, new-onset SLE patients were screened. Participants who met the inclusion criteria were randomly assigned (1:1) by the Department of Rheumatology and Immunology of Ruijin hospital to receive either MMF (MMF group) or control (Control group). Control group received treatment with HCQ (5mg/kg/day) and prednisone (0.5mg/kg/day); while MMF group received treatment with HCQ (5mg/kg/day) and prednisone (0.5mg/kg/day) plus MMF 500mg twice daily. The first follow-up of treatment was week 24, and then every 24 weeks for a total of 96 weeks. The primary endpoint was the proportion of SLE patients having flares (mild-to- moderate flare and severe flare) according to SELENA-SLEDAI Flare Index. The secondary endpoints included: 1) the proportion of lupus low disease activity state (LLDAS) at week 96; 2) short form-36 (SF-36) score before and after treatment; 3) the proportion of adverse events in the two groups during follow-up; 4) changes in SLEDAI-2000 score; 5) changes in prednisone dose.

INCLUSION CRITERIA

During enrolment, patients were required:
Inclusion Criteria:
1) Age between 18 and 65 years old;
2) Diagnosed with SLE at the time of screening by fulfilling ACR 2019 classified criteria or its 2017 version;
3) Have not received any prior SLE treatment;
4) Have a positive antinuclear antibody (HEp-2 titer ⩾1:80);
5) Have a positive anti-dsDNA antibody (fulfilled both anti-dsDNA (ELISA) ⩾ 300 IU/mL and anti-dsDNA (CLFT) ⩾1:10);
6) Do not have major organ involvement (i.e., brain, heart, liver, kidney, lung, muscle, serous cavity and gastrointestinal tract). Participants were permitted to have rash, arthritis, alopecia, oral ulcer, and mild hematologic system involvement (white blood cell >1.5*10^9/L and <4*10^9/L, hemaglobin>90g/L and <120g/L, platelet>60*10^9/L and <100*10^9/L).

EXCLUSION CRITERIA

Exclusion Criteria:
1) SLE patients who had been treated,
2) Liver and kidney dysfunction (Alanine aminotransferase (ALT)/ aspartate aminotransferase (AST) > 2 times upper normal limits; creatinine clearance rate < 60ml/min),
3) Cancer,
4) Recent infection or hematologic diseases not caused by SLE,
5) Pregnancy or planned to be pregnant,
6) Patients not willing to join the study.

RESEARCH CENTER/LOCATION

Ruijin Hospital, Shanghai Jiao Tong University School of Medicine;
The First Affiliated Hospital of Wenzhou Medical University, Wenzhou Medical University;
The Second Affiliated Hospital of Shandong First Medical University, Shandong First Medical University.

INTERVENTIONS/PROCEDURES

In this study, new-onset SLE patients were screened. Participants who met the inclusion criteria were randomly assigned (1:1) by the Department of Rheumatology and Immunology of Ruijin hospital to receive either MMF (MMF group) or control (Control group). Control group received treatment with HCQ (5mg/kg/day) and prednisone (0.5mg/kg/day); while MMF group received treatment

	with HCQ (5mg/kg/day) and prednisone (0.5mg/kg/day) plus MMF 500mg twice daily. The first follow-up of treatment was week 24, and then every 24 weeks for a total of 96 weeks. The primary endpoint was the proportion of SLE patients having flares (mild-to- moderate flare and severe flare) according to SELENA-SLEDAI Flare Index. The secondary endpoints included: 1) the proportion of lupus low disease activity state (LLDAS) at week 96; 2) short form-36 (SF-36) score before and after treatment; 3) the proportion of adverse events in the two groups during follow-up; 4) changes in SLEDAI-2000 score; 5) changes in prednisone dose.
**NUMBER OF PATIENTS**	We supposed the flare rate to be 10% in MMF group and 30% in Control group. Z test with unpooled variance (alpha=0.0568, beta=0.1964, power=0.8036) was used for sample size calculation. A sample size of 58 patients per group would provide the trial with 80% power at a two-sided alpha error of 0.05 to detect a difference between the two groups using the software PASS version 11.0. Taking an estimated 10% drop-out into account, a sample size of 65 patients per group could fulfill the statistical requirement.
**STUDY DURATION**	September 2018 to September 2021
**SUBJECT PARTICIPATION TIME**	96 weeks per subject from screening enrollment to completion of all follow-up visits

Ethics Committee of Ruijin Hospital affiliated Shanghai Jiao Tong University school of medicine

Application Form for Participation in Research

Date: 2018.08.09

Study title: A randomized controlled trial for the long-term outcomes of MMF on new onset SLE patients with high titers of anti-dsDNA antibody for reducing main organ involvement

Name and educational background of the principal investigator: Junna Ye, MD & PhD

Department: Rheumatology and Immunology

Tel: 13817366731

Email: yjn0912@qq.com

Names and educational qualifications of participating researchers: See follow-up list for details

If there is a research coordinator, the name and contact details are: None

Funding sources: National Natural Science Foundation of China (81801592), Shanghai Pujiang Young Rheumatologists Training Program (SPROG202006), Ruijin-Zhongmeihuadong Lupus Funding

I. Letter of Commitment

Principal Investigator:

I confirm that the participating researcher mentioned above has accepted his/her role in this research. I agree to continue to exchange information with the Ethics Committee regarding the protection of the rights of human research subjects, and agree to obtain the consent of the Ethics Committee before making any changes to the project. I will submit a progress report on the project at least once a year, or periodically as requested by the Ethics Committee. I agree to report to the Ethics Committee in a timely manner any unanticipated problems in the research or events that adversely affect human research subjects. An informed consent form will be issued to each study subject, which will be withdrawn after their signature and kept as a document. If the research involves the treatment of hospital patients, we will place a copy of the informed consent in the medical record of each research subject.

JUNNA YE — 2018.08.09

Principal Investigator Signature — Date

Head of clinical department (If the leader is the investigator or is not able to review, ask the deputy head to sign):

I have reviewed this research project and I believe it is reasonable. The design and methodology of the study are adequate to enable the purpose of the study to be achieved, and in addition the investigators have the appropriate financial and other resources. I support the conduct of this study and am therefore submitting it for further review.

CHENGDE YANG — Department of Rheumatology & Immunology — 2018.08.09

Department Head's Signature — Department — Date

II. Brief list

Does the research involve the following aspects?	YES	NO
Survey, Questionnaire or Interview	☒	☐
Existing medical records and/or samples	☒	☐
Investigational Drug IND# **Mycophenolate Mofetil**	☒	☐
Approved drugs for use in "non-CFDA approved" situations	☐	☒
Placebo	☐	☒
Test equipment, instruments, machines IDE#	☐	☒
Genetic research using samples from research subjects	☐	☒
Save samples of research subjects for future research when needed	☐	☒
Fetal tissue	☐	☒
Photo, audio and video recording	☐	☒
Involving non-patient volunteers	☐	☒
Patients as objects	☒	☐
Minors (under 18 years old)<br>If "Yes", indicate: Age range to years	☐	☒
You intend to recruit people who are:	☐	☒
-Neurologically damaged or mentally retarded?	☐	☒
-Prisoners, parolees and others convicted?	☐	☒
-Pregnant?	☐	☒
Want an HIV test?	☒	☐
Is this a multicenter study?	☒	☐
If "Yes", is Ruijin Hospital the initiator or coordinator?	☒	☐
Research using diagnostic or therapeutic ionizing radiation, or radioisotopes	☐	☒
DNA recombination or gene transplantation in human research subjects	☐	☒
Is this an oncology study?	☐	☒
Whether it involves the export of human samples	☐	☒
Whether foreign-funded enterprises are involved in project implementation (including	☐	☒

commissioned research, central laboratory testing, etc.)

III. Detailed description of research activities

1. Research abstract

1.1 Abstract

Research name: A randomized controlled trial for the long-term outcomes of MMF on new onset SLE patients with high titers of anti-dsDNA antibody for reducing main organ involvement

Research Introduction: Systemic lupus erythematosus (SLE) is an autoimmune disease that can affect multiple organs. The abnormality of the immune system is manifested by the existence of a large number of circulating self-antigens in the body, resulting in the production of autoantibodies against various self-antigens and causing immune damage to organs. Among them, anti-dsDNA antibodies are the most important autoantibodies, and high titers of anti-dsDNA antibodies are closely related to the pathogenesis of lupus nephritis, and can cause damage to other multiple organs, such as liver, heart, and lungs. Studies have shown that the incidence of SLE in the Asian population is about 8.4/100,000 people/year, and severe cases can even be life-threatening, bringing a significant economic burden to people's lives and economy. As a commonly used immunosuppressant, mycophenolate mofetil (MMF) has been widely used for induction and maintenance of lupus nephritis, and also plays an important role in the treatment of other extra-renal organ damage.

Therefore, we propose the scientific hypothesis that whether early application of MMF to newly diagnosed SLE patients with high titer of anti-dsDNA antibody and without major organ involvement could improve the long-term prognosis.

In this study, new-onset SLE patients were screened. Participants who met the inclusion criteria were randomly assigned (1:1) by the Department of Rheumatology and Immunology of Ruijin hospital to receive either MMF (MMF group) or control (Control group). Control group received treatment with HCQ (5mg/kg/day) and prednisone (0.5mg/kg/day); while MMF group received treatment with HCQ (5mg/kg/day) and prednisone (0.5mg/kg/day) plus MMF 500mg twice daily. The first follow-up of treatment was week 24, and then every 24 weeks for a total of 96 weeks. The primary endpoint was the proportion of SLE patients having flares (mild-to-moderate

flare and severe flare) according to SELENA-SLEDAI Flare Index. The secondary endpoints included: 1) the proportion of lupus low disease activity state (LLDAS) at week 96; 2) short form-36 (SF-36) score before and after treatment; 3) the proportion of adverse events in the two groups during follow-up; 4) changes in SLEDAI-2000 score; 5) changes in prednisone dose. This study is of great importance to improve the level of diagnosis and treatment in this field in China, and to promote Chinese clinical research results to the world, even into clinical guidelines, and to improve the academic level and status of China.

Research purposes:

This project mainly investigates whether early application of MMF to newly diagnosed SLE patients with high titer of anti-dsDNA antibody and without major organ involvement could improve the long-term prognosis.
Objective 1: The proportion of SLE patients having flares (mild-to-moderate flare and severe flare) according to SELENA-SLEDAI Flare Index.
Objective 2:
1) The proportion of lupus low disease activity state (LLDAS) at week 96;
2) Short form-36 (SF-36) score before and after treatment;
3) The proportion of adverse events in the two groups during follow-ups;
4) Changes in SLEDAI-2000 score;
5) Changes in prednisone dose.

Research objects:

A total of 130 people were enrolled, and they were randomly divided into the experimental group and the control group at a 1:1 ratio by randomization, with 65 cases in each group.
During enrolment, patients were required:
Inclusion Criteria:
1) Age between 18 and 65 years old;
2) Diagnosed with SLE at the time of screening by fulfilling ACR 2019 classified criteria or its 2017 version;
3) Have not received any prior SLE treatment;
4) Have a positive antinuclear antibody (HEp-2 titer ⩾1:80);
5) Have a positive anti-dsDNA antibody (fulfilled both anti-dsDNA (ELISA) ⩾ 300 IU/mL and anti-dsDNA (CLFT) ⩾1:10);
6) Do not have major organ involvement (i.e., brain, heart, liver, kidney, lung, muscle, serous cavity and gastrointestinal tract). Participants were permitted to have rash, arthritis, alopecia, oral ulcer, and mild hematologic system involvement (white blood cell >1.5*10^9/L and <4*10^9/L, hemaglobin>90g/L and <120g/L,

	platelet>60*10^9/L and <100*10^9/L).<br>Exclusion Criteria:<br>1) SLE patients who had been treated,<br>2) Liver and kidney dysfunction (Alanine aminotransferase (ALT)/ aspartate aminotransferase (AST) > 2 times upper normal limits; creatinine clearance rate < 60ml/min),<br>3) Cancer,<br>4) Recent infection or hematologic diseases not caused by SLE,<br>5) Pregnancy or planned to be pregnant,<br>6）Patients not willing to join the study.
**Research center/Location:**	Ruijin Hospital, Shanghai Jiao Tong University School of Medicine;<br>The First Affiliated Hospital of Wenzhou Medical University, Wenzhou Medical University;<br>The Second Affiliated Hospital of Shandong First Medical University, Shandong First Medical University.
**Research Interventions:**	In this study, new-onset SLE patients were screened. Participants who met the inclusion criteria were randomly assigned (1:1) by the Department of Rheumatology and Immunology of Ruijin hospital to receive either MMF (MMF group) or control (Control group). Control group received treatment with HCQ (5mg/kg/day) and prednisone (0.5mg/kg/day); while MMF group received treatment with HCQ (5mg/kg/day) and prednisone (0.5mg/kg/day) plus MMF 500mg twice daily. The first follow-up of treatment was at week 24, and then every 24 weeks for a total of 96 weeks. The primary endpoint was the proportion of SLE patients having flares (mild-to-moderate flare and severe flare) according to SELENA-SLEDAI Flare Index. The secondary endpoints included: 1) The proportion of lupus low disease activity state (LLDAS) at week 96; 2) Short form-36 (SF-36) score before and after treatment; 3) The proportion of adverse events in the two groups during follow-up; 4) Changes in SLEDAI-2000 score; 5) Changes in prednisone dose.
**Study Duration:**	September 2018 to September 2021
**Subject participation time:**	96 weeks per subject from screening enrollment to completion of all follow-up visits

1.2 Workflow

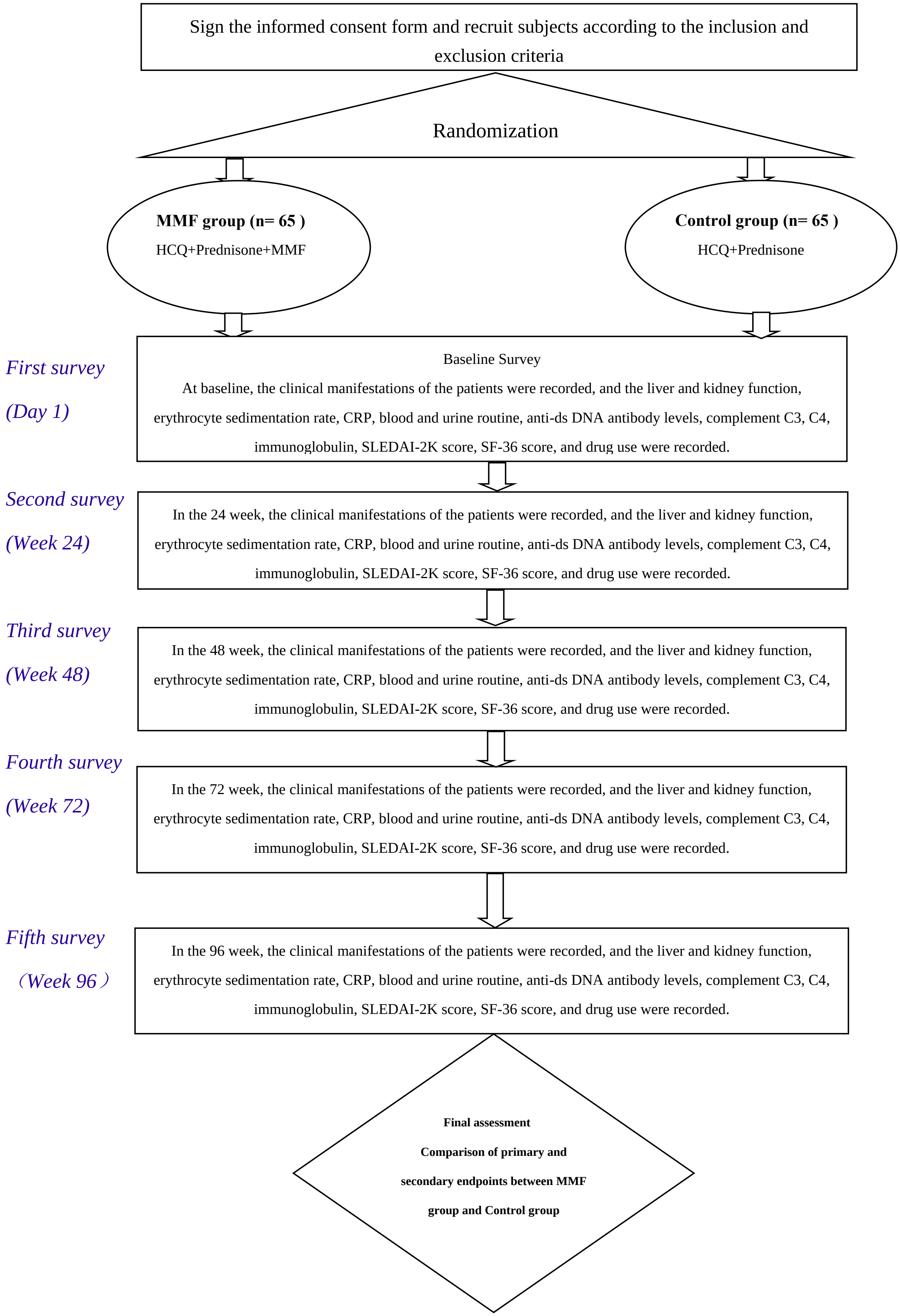

Sign the informed consent form and recruit subjects according to the inclusion and exclusion criteria
Randomization
MMF group (n= 65)
HCQ+Prednisone+MMF
Control group (n= 65)
HCQ+Prednisone
First survey
(Day 1)
Baseline Survey
At baseline, the clinical manifestations of the patients were recorded, and the liver and kidney function, erythrocyte sedimentation rate, CRP, blood and urine routine, anti-ds DNA antibody levels, complement C3, C4, immunoglobulin, SLEDAI-2K score, SF-36 score, and drug use were recorded.
Second survey
(Week 24)
In the 24 week, the clinical manifestations of the patients were recorded, and the liver and kidney function, erythrocyte sedimentation rate, CRP, blood and urine routine, anti-ds DNA antibody levels, complement C3, C4, immunoglobulin, SLEDAI-2K score, SF-36 score, and drug use were recorded.
Third survey
(Week 48)
In the 48 week, the clinical manifestations of the patients were recorded, and the liver and kidney function, erythrocyte sedimentation rate, CRP, blood and urine routine, anti-ds DNA antibody levels, complement C3, C4, immunoglobulin, SLEDAI-2K score, SF-36 score, and drug use were recorded.
Fourth survey
(Week 72)
In the 72 week, the clinical manifestations of the patients were recorded, and the liver and kidney function, erythrocyte sedimentation rate, CRP, blood and urine routine, anti-ds DNA antibody levels, complement C3, C4, immunoglobulin, SLEDAI-2K score, SF-36 score, and drug use were recorded.
Fifth survey
（Week 96）
In the 96 week, the clinical manifestations of the patients were recorded, and the liver and kidney function, erythrocyte sedimentation rate, CRP, blood and urine routine, anti-ds DNA antibody levels, complement C3, C4, immunoglobulin, SLEDAI-2K score, SF-36 score, and drug use were recorded.
Final assessment
Comparison of primary and secondary endpoints between MMF group and Control group

2. Research Background

2.1 Research Significance

This research will investigate the long-term prognosis and impact on organs in newly diagnosed SLE patients with high titer of anti-dsDNA antibody and without major organ involvement, in a randomized controlled trial with or without MMF. This research is important to improve the treatment in this field.

2.2 Research Background

Systemic lupus erythematosus (SLE) is an autoimmune disease that can affect multiple organs in the body, and is known as the prototype of autoimmune disease. The abnormality of the immune system is manifested by the existence of a large number of circulating self-antigens in the body, resulting in the production of a large number of autoantibodies against various self-antigens and causing immune damage to organs. Research shows that the incidence in Asian population is about 8.4/100,000 people/year[1]. SLE can affect multiple systems in the body, such as kidney, heart, and lung. In severe cases, it can be life-threatening and bring a significant economic burden to people's lives and economy. The current 10-year mortality rate of SLE patients in China reaches 20%[2].Therefore, early treatment of patients is particularly important. Evidence from clinical studies has revealed that many autoantibodies can appear earlier than the onset of the disease, and it has been reported that many autoantibodies, such as anti-nuclear, anti-dsDNA, and anti-Sm antibodies, can be detected in the serum of patients before the diagnosis of SLE disease, even 5 years before the first symptoms of SLE [3]. To date, hundreds of autoantibodies have been detected in SLE patients, most of which are useful in the diagnosis of the disease, and some of which are pathogenic and associated with lupus nephritis, such as anti-dsDNA and anti-C1q antibodies. The prevalence of nephritis in patients with high titers of anti-dsDNA antibodies was 30.2%, compared with only 18.7% in negative patients [4]. Anti-dsDNA antibodies are an independent risk factor for SLE disease activity score (SLEDAI score) greater than 5, which can lead to nephritis, arthritis and serositis[5]. Because patients who are not treated in the early stage of the disease often lead to multi-organ damage, even if they are actively treated in the late stage of the disease, they still cause irreversible severe consequences. Glucocorticoids use can reduce disease relapse in SLE patients with high titers of anti-dsDNA antibodies[6]. Glucocorticoids can reduce the expression of cytokines and adhesion molecules, inhibiting the tendency of leukocytes to enter sites of inflammation, and interfering with leukocyte, fibroblast, and endothelial cells. Glucocorticoids reduce the expression of MHC and Fc receptors and the synthesis of pro-inflammatory cytokines such as IL-2, IL-6, and TNF-α. Glucocorticoids also have an effect on T cells, reducing the production and action of IL-2 and the total number of circulating T cells. In addition, glucocorticoids also affect fibroblast function by decreasing vascular permeability, expression of adhesion molecules and IL-1 production. Finally, glucocorticoids affect fibroblast function by reducing fibroblast proliferation and the production of fibronectin and prostaglandins [7].This lead to the decline of the body's ability to resist infection, resulting in increased mortality, with prednisone ≤5 mg/d associated with

infection risk of 1.1 and greater than 20 mg/d associated with infection risk of 1.85[8]. Other side effects of glucocorticoids include increased blood glucose, blood pressure and osteoporosis. The advent of immunosuppressants has reduced the use of glucocorticoids and improved the survival rate of SLE patients. Mycophenolate mofetil (MMF) has been widely used as a commonly used immunosuppressant to induce remission and as a maintenance drug in lupus nephritis. MMF selectively inhibits the classical guanine synthesis pathway in lymphocytes by reversibly inhibiting hypoxanthine mononucleotide dehydrogenase without affecting the purine remediation synthesis pathway. Lymphocytes only have the classical synthesis pathway, but not the remediation synthesis pathway, so MMF can selectively inhibits lymphocyte proliferation by inhibiting the lymphocyte growth cycle, T cell and B cell proliferation and antibody production, as well as inhibiting glycoprotein conversion, reducing adhesion factor activity, inhibiting vascular smooth muscle cell proliferation and macrophage accumulation [9,10]. MMF is also effective in active SLE with extra-renal involvement and can lead to a reduction in the use of glucocorticoids[11]. The international expert group on SLE has identified key issues for future research in SLE, among which early treatment of SLE patients is one of the hot topics [12]. Although MMF can be useful for controlling disease activity, interventions that begin in the early stages of SLE before systemic involvement have not been reported. Whether the addition of early treatment with MMF in new onset SLE patients with high titers of anti-dsDNA antibodies but without systemic involvement can improve the long-term prognosis and reduce disease flare remains to be investigated.

In conclusion, this research explores the long-term prognosis and impact on target organs in newly diagnosed SLE patients with high titer of anti-dsDNA antibodies but without major organ involvement.

References:

1. Rees F, Doherty M, Grainge MJ, et al.The worldwide incidence and prevalence of systemic lupus erythematosus: a systematic review of epidemiological studies. Rheumatology (Oxford). 2017;56(11):1945-1961.doi: 10.1093/rheumatology/kex260
2.Mak A, CheungMW, ChiewH, et al. Global trend of survival and damage of systemic lupus erythematosus: meta-analysis and meta-regression of observational studies from the 1950s to 2000s. Semin Arthritis Rheum. 2012;41:830–9. doi: 10.1016/j.semarthrit.2011.11.002
3.Doria A, Iaccarino L, Ghirardello A, et al. Long-termprognosis and causes of death in systemic lupus erythematosus. Am J Med. 2006;119:700–6. doi:10.1016/j.amjmed.2005.11.034
4.Conti F, Ceccarelli F, Perricone C,et al.Systemic Lupus Erythematosus with and without Anti-dsDNA Antibodies: Analysis from a Large Monocentric Cohort.Mediators Inflamm. 2015;2015:328078. doi: 10.1155/2015/328078
5. Andrejevic S, Jeremic I, Sefik-Bukilica M, et al.Immunoserological parameters in SLE: high-avidity anti-dsDNA detected by ELISA are the most closely associated with the disease activity.Clin Rheumatol. 2013;32(11):1619-26. doi: 10.1007/s10067-013-2330-3
6. Bootsma H, Spronk P, Derksen R,et al.Prevention of relapses in systemic lupus erythematosus. Lancet. 1995;345(8965):1595-9. doi: 10.1016/s0140-6736(95)90114-0
7. Buttgereit F, Saag KG, Cutolo M et al. The molecular basis for the effectiveness, toxicity

and resistance to glucocorticoids:focus on the treatment of rheumatoid arthritis. Scand J Rheumatol. 2005;34(1):14-21. doi: 10.1080/03009740510017706.
8. Dixon WG, Kezouh A, Bernatsky S et al. The influence of systemic glucocorticoid therapy upon the risk of non-serious infection in older patients with rheumatoid arthritis: a nested case-control study. Ann Rheum Dis. 2011;70(6):956-60. doi: 10.1136/ard.2010.144741.
9. Nossent J, Raymond W, Kang A, et al. The current role for clinical and renal histological findings as predictor for outcome in Australian patients with lupus nephritis. Lupus.2018;27(11):1838-1846. doi: 10.1177/0961203318792361.
10. Park DJ, Kang JH, Lee KE, et al.Efficacy and safety of mycophenolate mofetil and tacrolimus combination therapy in patients with lupus nephritis: a nationwide multicentre study.Clin Exp Rheumatol.2019;37(1):89-96.
11. Yahya F, Jasmin R, Ng CT, et al. Open label randomized controlled trial assessing the efficacy of mycophenolate sodium against other conventional immunosuppressive agents in active systemic lupus erythematosus patients without renal involvement. Int J Rheum Dis. 2013;16(6):724-30. doi: 10.1111/1756-185X.12179.
12. van Vollenhoven RF, Mosca M, Bertsias G,et al. Treat-to-target in systemic lupus erythematosus: recommendations from an international task force. Ann Rheum Dis. 2014;73(6):958-67. doi: 10.1136/annrheumdis-2013-205139.

2.3 Expected Outcomes of the Research

This randomized controlled trial was conducted to investigate the need for early treatment in newly diagnosed SLE patients with high titer of anti-dsDNA antibody and without major organ involvement. One SCI paper is expected to be completed.

2.4 Risk/Benefit Assessment

2.4.1 Potential risks

Currently, mycophenolate mofetil and glucocorticoids are used as first-line drugs in systemic lupus erythematosus. The research has no psychological harm, no financial loss and no legal harm to the patients. No pain and bodily harm. During treatment, patients may develop infection due to the use of glucocorticoids and immunosuppressants, as well as gastrointestinal reactions and blood counts that may be influenced by mycophenolate mofetil.

2.4.2 Known potential benefit

The social benefits of this research may be to improve the prognosis of patients with new-onset SLE. The benefit to individual subject lies in the early control of disease progression, which may be helpful for the long-term prognosis of patients.

2.4.3 Potential Risk/Benefit Assessment

Currently, mycophenolate mofetil and glucocorticoids are used as first-line drugs in systemic lupus erythematosus. The study has no psychological harm, no financial loss and no legal harm to the patients. No pain and bodily harm. Both glucocorticoids and immunosuppressants have the risk of causing infection. The side effects of the drug can be monitored, and if the infection occurs, anti-infective treatment can also be administered in the hospital.

3. Principal Investigator Information

3.1 Principal investigator name, qualifications, contact information, brief introduction

The principal investigator of this study is Dr. Junna Ye. Dr. Junna Ye is currently a physician in the Department of Rheumatology and Immunology, mainly engaged in clinical research on systemic lupus erythematosus (SLE). From 2014 to 2015, she studied the design and statistical methods of randomized controlled trial at the University of Southampton (UK) through the National Studying Abroad Program and received a GCP training certificate. She is currently in charge of the SLE clinical database and specimen bank in the Department of Rheumatology and Immunology of Ruijin Hospital. Currently, Department of Rheumatology and Immunology at Ruijin Hospital has more than 800 cases of SLE blood specimens and more than 200 cases of new onset SLE, which has laid a solid foundation for clinical and basic research on SLE. At present, multi-level and multi-faceted clinical studies have been conducted on SLE, and a number of research articles have been published: clinical cases of SLE lymphoedema published in BMJ ; application of SLE classification criteria in newly diagnosed SLE patients published in Ann Rheum Dis ; anti-PD-1 antibody in SLE published in Arthritis Res Ther ;and on pregnancy in SLE patients combined with antiphospholipid syndrome published in Clinical Rheumatology ; validation of SLE classification criteria in Chinese population published in Lupus; and another new marker in SLE, anti-tyro3 antibody, was published in Journal of Immunology Research. Currently, she has published 21 SCI papers as first author or corresponding author. As the project leader, she is currently responsible for the National Natural Science Foundation of China ("Research on the mechanism of anti-RCP antibodies involved in the pathogenesis of systemic lupus erythematosus by affecting the phagocytosis of macrophages", 81801592) , the Shanghai Young Scientist Project (18YF1414100), Shanghai University Young Teacher Training Grant Program (82013011620002) and Shanghai Jiao Tong University Multidiscipline Project (YG2016QN60), Guangci Excellent Youth Program of Ruijin Hospital (GCQN-2017-B05). In addition, Dr Ye went to John Hopkins Rheumatology Department for clinical study.

3.2 Main participants

No	Name	Sex	Title	Whether GCP training	Roles in research
1	Junna Ye	Female	Physician	yes	PI
2	Chengde Yang	Male	Physician	yes	Senior rheumatologist of DSMB

3	Jialin Teng	Female	Physician	yes	Senior rheumatologist of DSMB
4	Yijun You	Female	MD student	yes	Statistician
5	Zhuochao Zhou	Female	Physician	yes	Statistician
6	Fan Wang	Female	MD student	yes	Research assistant
7	Jian Li	Male	Statistician	yes	Statistician
8	Honglei Liu	Male	Physician	yes	Responsible for patient inclusion and regular follow-up assessments
9	Xiaobing Cheng	Female	Physician	yes	Responsible for patient inclusion and regular follow-up assessments
10	Yutong Su	Female	Physician	yes	Responsible for patient inclusion and regular follow-up assessments
11	Xiaowei Chen	Female	Physician	yes	Responsible for patient inclusion and regular follow-up assessments
12	Hui Zheng	Female	Physician	yes	Responsible for patient inclusion and regular follow-up assessments
13	Jing Xu	Female	nephrologist	yes	Responsible for pathology of lupus nephritis

4. Research objectives

This project investigates whether early application of MMF to newly diagnosed SLE patients with high titer of anti-dsDNA antibody and without major organ involvement could improve the long-term prognosis.

Objective 1: The proportion of SLE patients having flares (mild-to-moderate flare and severe flare) according to SELENA-SLEDAI Flare Index.
Objective 2:
1) The proportion of lupus low disease activity state (LLDAS) at week 96;
2) Short form-36 (SF-36) score before and after treatment;
3) The proportion of adverse events in the two groups during follow-ups;
4) Changes in SLEDAI-2000 score;
5) Changes in prednisone dose.

The primary outcome was the proportion of patients who have flares (mild-to-moderate flares and severe flares) defined by modified SELENA-SLEDAI Flare Index (SFI) at 96 weeks of

follow-up.
Secondary Endpoints:
1) The proportion of lupus low disease activity state (LLDAS) at week 96;
2) Short form-36 (SF-36) score of before and after treatment in two groups;
3) The proportion of adverse events of two groups in the follow-ups;
4) Changes in SLEDAI-2000 score;
5) Changes in prednisone dose.

5. Research design

5.1 General design

This research is a randomized clinical trial with 65 patients in each group calculated according to the PASS 11.0 software. In this trial, new-onset SLE patients were screened. Participants who met the inclusion criteria were randomly assigned (1:1) to receive either MMF (MMF group) or control (Control group). Control group received treatment with HCQ (5mg/kg/day) and prednisone (0.5mg/kg/day); while MMF group received treatment with HCQ (5mg/kg/day) and prednisone (0.5mg/kg/day) plus MMF 500mg twice daily. The first follow-up of treatment was at week 24, and then every 24 weeks for a total of 96 weeks. The primary endpoint was the proportion of SLE patients having flares (mild-to-moderate flare and severe flare) according to SELENA-SLEDAI Flare Index. The secondary endpoints included: 1) The proportion of lupus low disease activity state (LLDAS) at week 96; 2) Short form-36 (SF-36) score before and after treatment; 3) The proportion of adverse events in the two groups during follow-up; 4) Changes in SLEDAI-2000 score; 5) Changes in prednisone dose.

5.2 Define research endpoints

The primary endpoint was the proportion of SLE patients having flares (mild-to-moderate flare and severe flare) according to SELENA-SLEDAI Flare Index. The secondary endpoints included: 1) The proportion of lupus low disease activity state (LLDAS) at week 96; 2) Short form-36 (SF-36) score before and after treatment; 3) The proportion of adverse events in the two groups during follow-up; 4) Changes in SLEDAI-2000 score; 5) Changes in prednisone dose.

5.3 Determining sample size

We referred to the flare rate of the placebo arm in 'Met Lupus' trial[1] and the flare rate from the BLISS-52, BLISS-76 and BLISS-North East Asia phase III trials[2,3]. We supposed the flare rate to be 10% in MMF group and 30% in Control group. Z test with unpooled variance (alpha=0.0568, beta=0.1964, power=0.8036) was used for sample size calculation. A sample size of 58 patients per group would provide the trial with 80% power at a two-sided alpha error of 0.05 to detect a difference between the two groups using the software PASS version 11.0. Taking an estimated 10% drop-out into account, a sample size of 65 patients per group could fulfill the statistical requirement. The sample size was calculated using PASS version 11.0 software as follows:

Two Independent Proportions (Null Case) Power Analysis
Numeric Results of Tests Based on the Difference: P1 - P2
H0: P1-P2=0. H1: P1-P2=D1<>0. Test Statistic: Z test with unpooled variance

Power	Sample Size Grp 1 N1	Sample Size Grp 2 N2	Prop|H1 Grp 1 or Trtmnt P1	Prop Grp 2 or Control P2	Diff if H0 D0	Diff if H1 D1	Target Alpha	Actual Alpha	Beta
0.8036	58	58	0.1000	0.3000	0.0000	-0.2000	0.0500	0.0568	0.1964

Reference:

1 Sun FF, Wang HJ, Liu Z, et al. Safety and efficacy of metformin in systemic lupus erythematosus: a multicentre, randomised, double-blind, placebo-controlled trial. Lancet Rheumatol. 2020;2:E210-E16.

2 Furie R, Petri M, Zamani O, et al. A phase III, randomized, placebo-controlled study of belimumab, a monoclonal antibody that inhibits B lymphocyte stimulator, in patients with systemic lupus erythematosus. Arthritis Rheum. 2011;63(12):3918-30. doi: 10.1002/art.30613.

3 Navarra SV, Guzman RM, Gallacher AE, et al. Efficacy and safety of belimumab in patients with active systemic lupus erythematosus: a randomised, placebo-controlled, phase 3 trial. Lancet. 2011;377(9767):721-31. doi: 10.1016/S0140-6736(10)61354-2.

6. Research object

6.1 Inclusion criteria

1) Age between 18 and 65 years old;
2) Diagnosed with SLE at the time of screening by fulfilling ACR 2019 classified criteria or its 2017 version;
3) Have not received any prior SLE treatment;
4) Have a positive antinuclear antibody (HEp-2 titer ⩾1:80);
5) Have a positive anti-dsDNA antibody (fulfilled both anti-dsDNA (ELISA) ⩾ 300 IU/mL and anti-dsDNA (CLFT) ⩾1:10);
6) Do not have major organ involvement (i.e., brain, heart, liver, kidney, lung, muscle, serous cavity and gastrointestinal tract). Participants were permitted to have rash, arthritis, alopecia,

oral ulcer, and mild hematologic system involvement (white blood cell >1.5*10^9/L and <4*10^9/L, hemaglobin>90g/L and <120g/L, platelet>60*10^9/L and <100*10^9/L).

6.2 Exclusion criteria

1) SLE patients who had been treated；
2) Liver and kidney dysfunction (Alanine aminotransferase (ALT)/ aspartate aminotransferase (AST) > 2 times upper normal limits; creatinine clearance rate < 60ml/min)；
3) Cancer；
4) Recent infection or hematologic diseases not caused by SLE；
5) Pregnancy or planned to be pregnant；
6) Patients not willing to join the study.

6.3 Things to pay attention to in life

None

6.4 Recruitment of patients

We referred to the flare rate of the placebo arm in ‘Met Lupus’ trial[1] and the flare rate from the BLISS-52, BLISS-76 and BLISS-North East Asia phase III trials[2,3]. We supposed the flare rate to be 10% in MMF group and 30% in Control group. Z test with unpooled variance (alpha=0.0568, beta=0.1964, power=0.8036) was used for sample size calculation. A sample size of 58 patients per group would provide the trial with 80% power at a two-sided alpha error of 0.05 to detect a difference between the two groups using the software PASS version 11.0. Taking an estimated 10% drop-out into account, a sample size of 65 patients per group could fulfill the statistical requirement.

Recruit patients according to the inclusion and exclusion criteria:
Inclusion Criteria:
1) Age between 18 and 65 years old;
2) Diagnosed with SLE at the time of screening by fulfilling ACR 2019 classified criteria or its 2017 version;
3) Have not received any prior SLE treatment;
4) Have a positive antinuclear antibody (HEp-2 titer ≥1:80);
5) Have a positive anti-dsDNA antibody (fulfilled both anti-dsDNA (ELISA) ≥ 300 IU/mL and anti-dsDNA (CLFT) ≥1:10);
6) Do not have major organ involvement (i.e., brain, heart, liver, kidney, lung, muscle, serous cavity and gastrointestinal tract). Participants were permitted to have rash, arthritis, alopecia, oral ulcer, and mild hematologic system involvement (white blood cell >1.5*10^9/L and <4*10^9/L, hemaglobin>90g/L and <120g/L, platelet>60*10^9/L and <100*10^9/L).

Exclusion Criteria:
1) SLE patients who had been treated ；
2) Liver and kidney dysfunction (Alanine aminotransferase (ALT)/ aspartate aminotransferase (AST) > 2 times upper normal limits; creatinine clearance rate < 60ml/min) ；
3) Cancer ；
4) Recent infection or hematologic diseases not caused by SLE, ；
5) Pregnancy or planned to be pregnant, ；
6) Patients not willing to join the study.

Control and grouping: computerized randomization method was used. Grouping was divided into Control and MMF group. Each patient signed an informed consent form before enrollment and the physician was obliged to answer the patient's questions during the follow-up, and the patient had the right to withdraw from the study at any time. Patients have the right to give consent if treatment changes occur.

Research centers:
Ruijin Hospital Affiliated to Shanghai Jiaotong University School of Medicine; The First Affiliated Hospital of Wenzhou Medical University; The Second Affiliated Hospital of Shandong First Medical University
Number of study subjects: 130 cases
Recruitment location: the above three hospitals
Measures to strengthen the compliance of research subjects: regularly follow up reminders to the patients.

6.5 Methods of subject assignment

Participants were randomly assigned (1:1) using blocks of four to receive either MMF (MMF group) or a control treatment (Control group). A statistician from the Clinical Trial Center of Ruijin Hospital, Li Jian, who was masked to trial allocation generated the randomization sequences by a computer algorithm. Then, random numbers were written on the cards and packed in sequentially numbered envelopes that were opened when the patients were enrolled. Dr. Junna Ye assigned patients to different groups according to the number on the card. Only Assessors were masked to the group allocation. Eligible subjects were recruited from the Department of Rheumatology and Immunology of Ruijin Hospital and the Department of Rheumatology and Immunology of the other two clinical centers.

7. Research interventions

7.1 Research interventions

7.1.1 Description of Intervention

Control group received treatment with HCQ (5mg/kg/day) and prednisone (0.5mg/kg/day); while MMF group received treatment with HCQ (5mg/kg/day) and prednisone (0.5mg/kg/day) plus MMF 500mg twice daily. Hydroxychloroquine is taken after meals and the manufacturer is Shanghai Pharmaceutical Co., Ltd, and the dosage form is tablets (100mg per tablet). The daily dose of mycophenolate mofetil is 500 mg twice daily, and the manufacturer is Hangzhou Zhongmei Huadong Pharmaceutical Co., Ltd., and the dosage form is capsule (250 mg per capsule).

Prednisone dose was tapered as follows: the initial dose was maintained for four weeks and then tapered 5mg every two weeks. When the dose reduced to 20 mg/day, it was tapered 5mg every month and then gradually to 0.1-0.2mg/kg/day. The taper of prednisone doses was determined by the physicians according to the assessment of the patients. Participants who met the criteria of severe flare were considered as having ended the trial and were treated with

increased doses of prednisone (more than 0.5mg/kg/day), immunosuppressive therapy or hospitalized for treatment according to the patients' condition and the physician's assessment.

7.1.2 Dosage and Administration

Control group received treatment with HCQ (5mg/kg/day) and prednisone (0.5mg/kg/day); while MMF group received treatment with HCQ (5mg/kg/day) and prednisone (0.5mg/kg/day) plus MMF 500mg twice daily. Hydroxychloroquine is taken after meals, the manufacturer is Shanghai Pharmaceutical Co., Ltd., and the dosage form is tablets (100mg per tablet). The daily dose of mycophenolate mofetil is 500 mg twice daily, the manufacturer is Hangzhou Zhongmei Huadong Pharmaceutical Co., Ltd., and the dosage form is capsules (250 mg per capsule). Prednisone dose was tapered as follows: the initial dose was maintained for four weeks and then tapered 5mg every two weeks. When the dose reduced to 20 mg/day, it was tapered 5mg every month and then gradually to 0.1-0.2mg/kg/day. The taper of prednisone doses was determined by the physicians according to the assessment of the patients. Participants who met the criteria of severe flare were considered as having ended the trial and were treated with increased doses of prednisone (more than 0.5mg/kg/day), immunosuppressive therapy or hospitalized for treatment according to the patients' condition and the physician's assessment.

7.1.3 Establishment, preservation, and unblinding methods of trial drug codes and methods of unblinding in emergency situations

Not applicable for this trial.

7.1.4 Items and times of clinical and laboratory examinations to be carried out

Patients were observed for clinical manifestations, assessment of disease condition, detection of liver and kidney function, erythrocyte sedimentation rate (ESR), CRP, blood and urine routine, anti-dsDNA antibody levels, complement C3, C4, immunoglobulin, SLEDAI-2K score, SF-36 score, medication use, and adverse reaction (AEs) records.

Number of visits: at screening + 4 follow-up visits (week 24, 48, 72, 96).

7.2 Preparation/handling/storage/responsibility

7.2.1 Responsibility

The drugs involved in this study were purchased by the patients from the outpatient pharmacy, and each time the dosage was prescribed according to the doctor's prescription.

7.2.2 Composition, Appearance, Packaging and Labeling

Prednisone: The manufacturer is Zhejiang Xianju Pharmaceutical, and the dosage form is tablet (5 mg per tablet);

Hydroxychloroquine: The manufacturer is Shanghai Pharmaceuticals, and the dosage form is tablet (100 mg per tablet).

Mycophenolate mofetil: The manufacturer is Hangzhou Zhongmei Huadong Pharmaceutical Co., Ltd., and the dosage form is capsule (250 mg per capsule).

7.2.3 Product Storage and Stability

Stored at room temperature and protected from light.

7.2.4 Preparation

The intervention drugs are fixed-package tablets and capsules, which require no additional preparation.

7.3 Measures to reduce bias: randomization and blinding

Participants were randomly assigned (1:1) using blocks of four to receive either MMF (MMF group) or a control treatment (Control group). A statistician from the Clinical Trial Center of Ruijin Hospital, Li Jian, who was masked to trial allocation generated the randomization sequences by a computer algorithm. Then, random numbers were written on the cards and packed in sequentially numbered envelopes that were opened when the patients were enrolled in the trial. They were assigned to the trial group according to the number on the card. Eligible subjects were recruited from the Department of Rheumatology and Immunology of Ruijin hospital and Department of Rheumatology and Immunology of the other two clinical centers. Dr. Junna Ye randomly assigned patients to different groups according to number sequence. Assessors were masked to the group allocation. Observers were masked to the group allocation and they were responsible for regular follow-up assessments.Data analysts (Yijun You and Zhuochao Zhou) were not involved in patient assessment and treatment, and they were masked to the group allocation.

7.4 Follow-up and compliance

During the study, the patients were followed up for 4 times (week 24, week 48, week 72, week 96). Patients were observed for clinical manifestations, assessment of disease condition, detection of liver and kidney function, erythrocyte sedimentation rate (ESR), CRP, blood and urine routine, anti-dsDNA antibody levels, complement C3, C4, immunoglobulin, SLEDAI-2K score, SF-36 score, medication use, and adverse reaction (AEs) records at each follow-up visit time. Measures to strengthen the compliance of research subjects: regularly follow up reminders to the patients.

7.5 Research Intervention Commitment

Remind patients to follow up regularly and keep detailed records of each medication administered to patients, and check patients' medical records regularly.

7.6 Research plan

Time / Content	Screening period	Follow-up period			
	Screening day	Week 24	Week 48	Week 72	Week 96
Informed consent form	√				
Inclusion/Exclusion Criteria	√				
Physical examination	√	√	√	√	√
SLEDAI-2000 Index	√	√	√	√	√
Blood routine examination	√	√	√	√	√
Urine routine examination	√	√	√	√	√
ALT&AST	√	√	√	√	√
Scr	√	√	√	√	√
ESR & CRP	√	√	√	√	√

Complement 3 & 4	√	√	√	√	√
Anti-dsDNA antibody	√	√	√	√	√
Immunoglobulin G	√	√	√	√	√
SF-36 score	√	√	√	√	√
Medication	√	√	√	√	√
Adverse events	√	√	√	√	√

8. Study Intervention Discontinuation and Study Subject Discontinuation/Withdrawal

8.1 Research Intervention Discontinuation

1) Patients who met the criteria of severe flare were considered as having ended the trial. Patients with severe flare were not eligible for the current treatment and need to be further evaluated by assessors, it may be necessary for them to discontinue study intervention, the patient will remain in the study to be evaluated for the remainder of the assessment visits.
2) Patients who met the criteria of severe flare were treated with increased doses of prednisone (more than 0.5mg/kg/day), immunosuppressive therapy or hospitalized for treatment according to the patients' condition and the physician's assessment.
3) Patients with mild-to-moderate flares continued to be followed up till the end of Week 96, and all flare episodes were recorded.
4) Patients with mild-to-moderate flares could increase the dose of prednisone (less than 0.5mg/kg/day) or nonsteroidal anti-inflammatory drugs.
5) For fever, rash, and arthritis, the dose of prednisone could be used at an initial dose of less than 0.5mg/kg/day, and following the assessment of symptoms, the dose could be increased to more than 0.5mg/kg/day if needed.
6) Any sign of kidney involvement (glomerular hematuria and/or cellular casts, proteinuria >0.5g/24hours (or spot urine protein-to-creatine ratio (UPCR) >500mg/g), unexplained decrease in glomerular filtration rate (GFR)), renal biopsy was suggested to confirm the diagnose of LN.
7) The treatment of LN was provided according to the European Renal Association-European Dialysis and Transplant Association (ERA-EDTA) guideline.
8) The treatment of other organ involvement was provided following the 2019 European League Against Rheumatism (EULAR) recommendation.
9) Other situations:
a) Liver and kidney dysfunction (Alanine aminotransferase (ALT)/ aspartate aminotransferase (AST) > 2 times upper normal limits; creatinine clearance rate < 60ml/min),
b) Cancer,
c）Recent infection or hematologic diseases not caused by SLE,
d）Pregnancy or planned to be pregnant,
e）Patients not willing to join the study.

Other situations including liver or kidney dysfunction, cancer, recent infections or hematologic diseases not caused by SLE, pregnancy or planning to be pregnant, patient will also remain in the study to be evaluated for the remainder of the assessment visits.

8.2 Subject discontinuation/withdrawal

Patients may withdraw from the study at any time at his/her own request. Patients could withdraw at the discretion of the investigator for safety, behavioral, or compliance reasons. The patient will be permanently discontinued from the study intervention and the study at that time. If the participant withdraws informed consent, future information will stop collecting, investigators may retain and continue to use any data collected before withdrawing of the consent.

8.3 Lost to follow-up

The follow-up period of this research is 96 weeks, and every 24 weeks is a follow-up interval. When the research subject stops the scheduled follow-up, fails to complete the prescribed procedures of the research, or the investigator cannot contact the research subject regarded as lost to follow-up. A participant will be considered lost to follow-up if he/she repeatedly fails to return for scheduled visits and is unable to be contacted by the study site.

9. Evaluation of research outcomes

9.1 Primary and secondary outcome evaluation

The primary outcome was the proportion of patients who have flares (mild-to-moderate flares and severe flares) defined by modified SELENA-SLEDAI Flare Index (SFI) at 96 weeks of follow-up.

Secondary Endpoint:

1) The proportion of lupus low disease activity state (LLDAS) at week 96.
2) Short form-36 (SF-36) score of before and after treatment in two groups.
3) The proportion of adverse events of two groups in the follow-ups.
4) Changes in SLEDAI-2000 score.
5) Changes in prednisone dose.

9.2 Safety and other evaluations

Safety indicators: clinical manifestations and signs of the patient, laboratory tests related to infection, laboratory tests related to SLE activity, such as complement, erythrocyte sedimentation rate (ESR), anti-dsDNA antibody titer and so on. Adverse events were reported regularly.

9.3 Adverse events and serious adverse events

9.3.1 Adverse Event (AE) Definition

According to ICH-GCP definition

An adverse medical event that occurs in a patient or clinical trial subjects following the use of a drug, but for which there is not necessarily a causal relationship between this medical event and treatment. Thus, an adverse event may be any adverse and unintended sign (including abnormal laboratory findings), symptom, or disease temporally associated with the use of a (study) drug,

regardless of whether it is related to the (study) drug. Currently, mycophenolate mofetil and glucocorticoids are used as first-line drugs in systemic lupus erythematosus. Infection could be considered as the most common AE of both glucocorticoids and immunosuppressants.

9.3.2 Serious Adverse Event (SAE) Definition

Adverse medical events at any dose as defined by ICH-GCP:
Cause death；
Life threatening；
Requiring hospitalization or prolonged hospitalization；
Resulting in permanent or severe disability/incapacity；
Congenital malformations/birth defects.

9.3.3 Adverse event classification

9.3.3.1 Incident Severity

Classified according to the Common Terminology Criteria for Adverse Events (CTCAE) version 5.0：
CTCAE provides a specific clinical description of the severity of each adverse event (Grade 1 to 5) based on the following basic guidelines.
Grade 1: Mild; asymptomatic or mild; seen clinically or diagnostically only; no treatment required
Grade 2: Moderate; requires lesser, local or non-invasive treatment; age-appropriate instrumental limitation of activities of daily living.
Grade 3: Severe or medically significant but not immediately life-threatening; results in hospitalization or prolonged hospitalization; disability; autonomic limitation of activities of daily living.
Grade 4: Life-threatening; requires urgent treatment.
Grade 5: Death related to AE.

9.3.3.2 Relevance to Research Interventions

Classified by determined relevant, possibly relevant, potentially relevant, unlikely relevant, irrelevant.

9.3.3.3 Expectancy

Adverse events are first identified as expected or unanticipated. If an adverse event is expected it should be collected using a standard format and its expectedness can be assessed based on previous observed adverse events; if the nature, severity, or frequency of the adverse event does not match the risk information described in previous study interventions, it can be considered unanticipated.

9.3.4 Timing, frequency, follow-up, and regression of adverse event assessments

The nature of the adverse event was determined by combining the definitions of AE and SAE from the ICH-GCP and GCP. All AEs including local and systemic reactions should be collected using a case report form (CRF) that collects information including description of the event, time of occurrence, physician's assessment of severity, relationship to the investigational product, and time to resolution/stabilization of the event. All AEs should be given adequate resolution. At each study follow-up visit, the investigator will ask about AEs/SAEs that have occurred since the

last follow-up visit, and the AE/SAE will be followed until resolved or stabilized.

9.3.5 Adverse Event Reporting

Describe the AE reporting process, including timeframes, according to relevant guidelines.

9.3.6 Serious Adverse Event Reporting

Describe SAE reporting procedures, including timeframes, according to relevant guidelines. The investigator will immediately report any SAE to the sponsor, whether or not it is related to the study intervention.

9.3.7 Reporting adverse events to study subjects

The information would be shared with subjects.

9.4 Unexpected problem

9.4.1 Definition of Unexpected Problems

Unexpected problems include events, experiences, or outcomes in which the study subjects meet the following criteria:

1) The problem is unintended in nature, severity, and frequency based on the study protocol, informed consent form, and characteristics of the study subject.

2) The study participant is at a more serious risk than previously recognized events, experiences, or outcomes that meet the definition of an unintended problem that should be guaranteed to be changed to protect the safety, welfare, or rights of the subjects.

Actions taken include:

1) Modifying inclusion and exclusion criteria to reduce newly identified risks

2) Implementation of additional safety monitoring procedures

3) Suspending recruitment of new subjects or discontinuing study procedures

4) Modifying the informed consent form to include a description of newly identified risks

5) Providing additional information on newly identified risks to subjects already enrolled in the study

9.4.2 Reporting of unexpected problems

Unanticipated problems should be reported as soon as possible and their reporting procedures described, including time frames.

Include the following information:

1) Detailed description of the event, experience, and ending

2) Explanation of the decision that the event, experience, or ending is an unintended problem

3) Actions to be taken to deal with the problem

10. Statistical analysis

10.1 General method

Random numbers were generated by computer. SPSS software (version 23) was used for data statistics, and PASS software (version 11.0) was used for sample size calculation. In descriptive statistics, data were expressed in the form of frequency (percentages) for categorical variables and medians (interquartile) or means ± standard deviations (SD) for continuous variables. In addition, t-tests were used to compare between-group differences in continuous variables after

exploring the normality of data distribution using the Shapiro-Wilk test. Mann-Whitney U tests were used to compare continuous variables with a skewed distribution. Pearson Chi-square or Fisher's exact tests were used to compare between-group differences in categorical variables. All tests were performed two-sided at the significance level of 5%.

10.2 Analysis of primary and secondary research endpoints

In descriptive statistics, data were expressed in the form of frequency (percentages) for categorical variables and medians (interquartile) or means ± standard deviations (SD) for continuous variables. In addition, t-tests were used to compare between-group differences in continuous variables after exploring the normality of data distribution using the Shapiro-Wilk test. Mann-Whitney U tests were used to compare continuous variables with a skewed distribution. Pearson Chi-square or Fisher's exact tests were used to compare between-group differences in categorical variables. Relative risk (RR) was used to compare the proportion of patients with the endpoint between the MMF and Control groups. We estimated the severe flare-free survival proportion of the MMF group and Control group using the Kaplan-Meier method with a log rank test. For the primary outcome assessed at two levels (mild-to-moderate and severe flares), Bonferroni correction was used and two-sided at a significance level of 0.025. Other tests were performed two-sided at the significance level of 0.05.

10.3 Security Analysis

AEs are coded and calculated and may be expressed using severity, frequency, and association with the intervention. Adverse events leading to discontinuation of the research intervention and serious AEs resulting from treatment should be listed individually.

10.4 Baseline Descriptive Analysis

Descriptive statistics were used to compare demographic characteristics and laboratory indicators between the groups at baseline.

10.5 Subgroup analysis

/

11. Supporting Documents and Notices

11.1 Informed Consent Process

Informed consent should be completed before the subject agrees to participate in the research and should continue throughout the research. The informed consent form was approved by the ethics committee, and the research subjects should read the informed consent form. The investigator will explain the research process and answer questions from the subject; and inform the subject of the possible risks and their rights. Subjects may discuss this with their families or guardians prior to consenting to participate.

The investigator must inform the subject that participation in the research is voluntary and that they may withdraw from the follow-up at any time during the trial. A copy of the informed consent form can be provided to the research subjects for preservation. The rights and welfare of

the research subjects will be protected and it is emphasized that the quality of their medical care will not be compromised by refusal to participate in the study.

11.2 Research Termination and Closure

Listing possible reasons for research discontinuation or suspension (unintended risks, failure to execute the study plan, quality of data and so on). In case of research suspension or moratorium, the person in charge should promptly notify the research subjects and report to the Ethics Committee and the Science and Technology Development Office. If the reasons for study discontinuation or suspension are resolved, the study can be resumed.

11.3 Privacy protection

This research involves the application of a dedicated person for the collection of specimens that need to be collected, and a dedicated person for the collection and storage of follow-up data. Only the research sponsor and relevant research personnel have access to the information, and any research information cannot be disclosed to unauthorized third parties without prior approved consent.

11.4 Collection and use of specimens and data

Only collected specimens and data for this study.

11.5 Quality Control and Quality Assurance

1) The physicians, nurses, doctoral students, clinical trial observation and other staff involved in this trial must have professional clinical knowledge as required in the trial protocol.
2) The investigator and other staff involved in the trial should perform their duties and strictly follow the clinical trial protocol and adopt standard operating procedures to ensure the implementation of the quality control and quality assurance system of the trial. The investigator and other staff involved in the study have obtained GCP certificate and have experience to control the quality of the study.
3) All observations and findings in clinical trials should be verified. Quality control must be performed at each stage of data processing to ensure the integrity, accuracy, authenticity and reliability of the data.
4) The investigator and other staff involved in the research had sufficient time to conduct the trial and a reliable source of subjects.
5) All items involving imaging and laboratory tests should comply with national standards.
6) This trial involves specimens that need to be collected should be collected by a dedicated person, and follow-up data should be collected and kept by a dedicated person.
7) The test procedures should be performed according to standard procedures.
8) When the research protocol needs to be modified, an ethics committee should be convened to give full play to the functions of the ethics committee and to ensure that the interests of the subjects are protected.
9) Save all the original data according to the requirements in the plan, in order of time for verification.
10) The contract research organization must assign supervisors who have been trained in this trial. The inspector must have a relevant professional background in medical pharmacology and strictly follow the standard process to verify the project.

11) The auditors systematically check the activities and documents related to the clinical trial to evaluate whether the trial is conducted in accordance with the trial protocol and standard operating procedures and relevant regulations, and whether the experimental data are timely, true, accurate and full record. The audit should be performed by personnel who are not involved in the clinical trial.
12) Audits are established to ensure that clinical trials are conducted in a manner consistent with standard operating procedures and relevant regulations.
13) Regular audits and meetings with relevant personnel to discuss issues found during audits.

11.6 Data processing and record keeping

11.6 Data collection and management

The types of source data to be collected in this trial are: medical records, AEs, questionnaires, laboratory results and so on. Data collection will be performed by clinical research staff under the supervision of the person in charge, who will be responsible for the accuracy, completeness, and timeliness of the reported data. All data should be clear to ensure accurate interpretation and to ensure their traceability. Clinical data will be maintained in a database, which should be password protected, and a logical proofreading process should be established when the database is created.

11.7 Publishing and Data Sharing Agreements

Data for all subjects included in the article, the research protocol and statistical analysis plan are available upon publication of the relevant results by sending an email inquiry to the research PI and obtaining consent from the research PI.

11. 8 Conflict of Interest Statement

None

12 Appendix

12.1 Workflow of glucocorticoids decrease process from baseline to week 96

In this trial, we randomly assigned patients (1:1) to Control group and MMF group. The Control group received treatment with HCQ (5mg/kg/day) and prednisone (0.5mg/kg/day); while MMF group received treatment with HCQ (5mg/kg/day) and prednisone (0.5mg/kg/day) plus MMF 500mg twice daily. The dose of MMF stayed the same unless a contrary or severe adverse event occurred.
Prednisone dose was tapered as follows:

Initial dose (0.5mg/kg/day) maintained for four weeks

↓

tapered 5mg every two weeks till to 20 mg/day

↓

tapered 5mg every month

↓

gradually to 0.1-0.2mg/kg/day

The taper of prednisone doses was determined by the physicians according to the assessment of

patients.

12.2 SF-36 questionnaire

12.2.1 SF-36 questionnaire Chinese version

SF-36 量表

请用画圈"√"的形式，选择符合您目前状态的内容。谢谢您的配合！

1、总体来讲，您的健康状况是：

①非常好　　②很好　　③好　　④一般　　⑤差

2、跟 1 年以前比您觉得自己的健康状况是：

①比 1 年前好多了　　②比 1 年前好一些　　③跟 1 年前差不多

④比 1 年前差一些　　⑤比 1 年前差多了

健康和日常活动

3、以下这些问题都和日常活动有关。请您想一想，您的健康状况是否限制了这些活动？如果有限制，程度如何？

（1）重体力活动。如跑步举重、参加剧烈运动等：

①限制很大　　②有些限制　　③毫无限制

（2）适度的活动。如移动一张桌子、扫地、打太极拳、做简单体操等：

①限制很大　　②有些限制　　③毫无限制

（3）手提日用品。如买菜、购物等：

①限制很大　　②有些限制　　③毫无限制

（4）上几层楼梯：

①限制很大　　②有些限制　　③毫无限制

（5）上一层楼梯：

①限制很大　　②有些限制　　③毫无限制

（6）弯腰、屈膝、下蹲：
①限制很大　②有些限制　③毫无限制

（7）步行 1500 米以上的路程：
①限制很大　②有些限制　③毫无限制

（8）步行 1000 米的路程：
①限制很大　②有些限制　③毫无限制

（9）步行 100 米的路程：
①限制很大　②有些限制　③毫无限制

（10）自己洗澡、穿衣：
①限制很大　②有些限制　③毫无限制

4、在过去 4 个星期里，您的工作和日常活动有无因为身体健康的原因而出现以下这些问题？
（1）减少了工作或其他活动时间：
①是　②不是

（2）本来想要做的事情只能完成一部分：
①是　②不是

（3）想要干的工作或活动种类受到限制：
①是　②不是

（4）完成工作或其他活动困难增多（比如需要额外的努力）：
①是　②不是

5、在过去 4 个星期里，您的工作和日常活动有无因为情绪的原因（如压抑或忧虑）而出现以下这些问题？
（1）减少了工作或活动时间：
①是　②不是

（2）本来想要做的事情只能完成一部分：
①是　②不是

（3）干事情不如平时仔细：
①是　②不是

6、在过去 4 个星期里，您的健康或情绪不好在多大程度上影响了您与家人、朋友、邻居或集体的正常社会交往？
①完全没有影响　②有一点影响　③中等影响　④影响很大　⑤影响非常大

7、在过去 4 个星期里，您有身体疼痛吗？
①完全没有疼痛　②有一点疼痛　③中等疼痛　④严重疼痛　⑤很严重疼痛

8、在过去 4 个星期里，您的身体疼痛影响了您的工作和家务吗？

①完全没有影响　②有一点影响　③中等影响　④影响很大　⑤影响非常大

您的感觉

9、以下这些问题是关于过去 1 个月里您自己的感觉，对每一条问题所说的事情，您的情况是什么样的？

（1）您觉得生活充实：

①所有的时间　②大部分时间　③比较多时间　④一部分时间

⑤小部分时间　⑥没有这种感觉

（2）您是一个敏感的人：

①所有的时间　②大部分时间　③比较多时间　④一部分时间

⑤小部分时间　⑥没有这种感觉

（3）您的情绪非常不好，什么事都不能使您高兴起来：

①所有的时间　②大部分时间　③比较多时间　④一部分时间　⑤小部分时间　⑥没有这种感觉

（4）您的心理很平静：

①所有的时间　②大部分时间　③比较多时间　④一部分时间

⑤小部分时间　⑥没有这种感觉

（5）您做事精力充沛：

①所有的时间　②大部分时间　③比较多时间　④一部分时间

⑤小部分时间　⑥没有这种感觉

（6）您的情绪低落：

①所有的时间　②大部分时间　③比较多时间　④一部分时间

⑤小部分时间　⑥没有这种感觉

（7）您觉得筋疲力尽：

①所有的时间　②大部分时间　③比较多时间　④一部分时间

⑤小部分时间　⑥没有这种感觉

（8）您是个快乐的人：

①所有的时间　②大部分时间　③比较多时间　④一部分时间

⑤小部分时间　⑥没有这种感觉

（9）您感觉厌烦：

①所有的时间　　②大部分时间　　③比较多时间　　④一部分时间

⑤小部分时间　⑥没有这种感觉

10、不健康影响了您的社会活动（如走亲访友）：

①所有的时间　　②大部分时间　　③比较多时间　　④一部分时间

⑤小部分时间　⑥没有这种感觉

总体健康情况

11、请看下列每一条问题，哪一种答案最符合您的情况？

（1）我好象比别人容易生病：

①绝对正确　②大部分正确　③不能肯定　④大部分错误　⑤绝对错误`

（2）我跟周围人一样健康：

①绝对正确　②大部分正确　③不能肯定　④大部分错误　⑤绝对错误

（3）我认为我的健康状况在变坏：

①绝对正确　②大部分正确　③不能肯定　④大部分错误　⑤绝对错误

（4）我的健康状况非常好：

①绝对正确　②大部分正确　③不能肯定　④大部分错误　⑤绝对错误

12.2.2 SF-36 questionnaire

SF-36 QUESTIONNAIRE

(1992 -- Medical Outcomes Trust)

Patient Name: ____________________　　　**Date:** ____________________

1. In general, would you say your health is: (circle one)

Excellent Very good Good Fair Poor

2. Compared to one year ago, how would you rate your health in general now? (circle one)

Much better now than one year ago.

Somewhat better now than one year ago.

About the same as one year ago.

Somewhat worse than one year ago.

Much worse than one year ago.

3. The following items are about activities you might do during a typical day. Does your health now limit you in these activities? If so, how much? (Mark each answer with an **X**)

**ACTIVITIES**	**Yes, limited a lot**	**Yes, limited a little**	**No, not limited at all**
a. **Vigorous activities,** such as running, lifting heavy objects, participating in strenuous sports			
b. **Moderate activities,** such as moving a table, pushing a vacuum cleaner, bowling, or playing golf			
c. Lifting or carrying groceries			
d. Climbing **several** flights of stairs			
e. Climbing **one** flight of stairs			
f. Bending, kneeling or stooping			
g. Walking **more than a mile**			
h. Walking **several blocks**			
i. Walking **one block**			
j. Bathing or dressing yourself			

4. During the past 4 weeks, have you had any of the following problems with your work or other regular daily activities as a result of your physical health? (Mark each answer with an **X**)

	**YES**	**NO**
a. Cut down on the **amount of time** you spent on work or other activities		
b. **Accomplished less** than you would like		
c. Were limited in the **kind** of work or other activities		
d. Had **difficulty** performing the work or other activities (for example, it took extra effort)		

5. During the past 4 weeks, have you had any of the following problems with your work or other regular daily activities as a result of any emotional problems (such as feeling depressed or anxious)? (Mark each answer with an **X**)

	**YES**	**NO**
a. Cut down the **amount of time** you spent on work or other activities		
b. **Accomplished less** than you would like		
c. Didn't do work or other activities as **carefully** as usual		

6.During the past 4 weeks, to what extent has your physical health or emotional problems interfered with your normal social activities with family, friends, neighbors or groups? (circle one)

Not at all Slightly Moderately Quite a bit Extremely

7.How much bodily pain have you had during the past 4 weeks? (circle one)

None Very mild Mild Moderate Severe Very severe

8.During the past 4 weeks, how much did pain interfere with your normal work (including both work outside the home and housework)?

Not at all A little bit Moderately Quite a bit Extremely

9. These questions are about how you feel and how things have been with you during the past 4 weeks. For each question, please give the one answer that comes closest to the way you have been feeling. How much of the time during the past 4 weeks (Mark each answer with an X)

	**All of the time**	**Most of the time**	**A Good Bit of the Time**	**Some of the time**	**A little of the time**	**None of the time**
a. Did you feel full of pep?						
b. Have you been a very nervous person?						
c. Have you felt so down in the dumps that nothing						
d. Have you felt calm and peaceful?						
e. Did you have a lot of energy?						
f. Have you felt downhearted and blue?						
g. Did you feel worn out?						
h. Have you been a happy person?						
i. Did you feel tired?						

10. During the past 4 weeks, how much of the time has your physical health or emotional problems interfered with your social activities (like visiting with friends, relatives, etc.)? (circle one)

All of the time　　Most of the time　　Some of the time　　A little of the time

None of the time

11.How TRUE or FALSE is each of the following statements for you?

	**Definitely true**	**Mostly true**	**Don't know**	**Mostly false**	**Definitely false**
a. I seem to get sick a little easier than other people					
b. I am as healthy as anybody I know					

c. I think my health is getting worse					
d. My health is excellent					

Mycophenolate mofetil and new-onset systemic lupus erythematosus with high titer of anti-dsDNA antibody and without major organ involvement: A Randomized Clinical Trial

STATISTICAL ANALYSIS PLAN

Version 2.0

Principal Investigator:

Junna Ye, MD & PhD

Department of Rheumatology and Immunology, Ruijin Hospital,

Shanghai Jiao Tong University School of Medicine

No. 197 Ruijin Second Road, Huangpu District, Shanghai 200025, China.

Tel. : +86 21 64370045 ;

Fax : +86 21 34186000 ;

F-mail : yjn0912@qq.com

TABLE OF CONTENTS

1. List of abbreviations and definition of terms 43
2. Introduction and background 45
3. Study outcomes 7
  3.1 Primary Endpoint 7
  3.2 Secondary Endpoint 48
4. Study design 48
5. Participant selection 50
  5.1 Inclusion Criteria 10

5.2 Exclusion Criteria 50
6. Sample size calculation 50
7. Analysis population 51
8. Statistical procedures 52
8.1 Randomization and masking 12
8.2 Analysis principles 52
8.3 Data quality control 53
8.4 Patients characteristics and baseline comparisons 53
8.5 Primary outcome 53
8.6 Secondary outcomes 54
8.7 Other variables 55
9. Flow chart 55
10. Statistical softwares 55
11. Study centers list 55
12. Study content schedule 55
13. Ethics 56
14. Adverse events disposition 56
15. Tables and figures 56
Figure 1 Flowchart 57
Table 1 Baseline characteristics 57
Table 2 MMF efficacy versus prednisone at visit xx 20
Table 3 Outcomes of 96-week follow-ups 21
Table 4 Adverse events 61
Table 5 The comparison of SF-36 scores before and after treatment 62
Table 6 The comparison of organ damage according to The Systemic Lupus International Collaborating Clinics (SLICC)/American College of Rheumatology (ACR) Damage Index (SDI) for SLE 24

1. List of abbreviations and definition of terms

ACR	American College of Rheumatology
AE	Adverse event
ALT	Alanine aminotransferase
AST	Aspartate aminotransferase
BP	Bodily pain
CI	Confidence intervals
CLIFT	Crithidia luciliae immunofluorescence test
CRF	Case report form
CRP	C-Reactive Protein
CTCAE	Common Terminology Criteria for Adverse Events
DMARDs	disease-modifying antirheumatic drugs

ELISA	Enzyme-linked immunosorbent assay
ERA-EDTA	European Renal Association-European Dialysis and Transplant Association
ESR	Erythrocyte sedimentation rate
EULAR	European League Against Rheumatism
GCP	Good Clinical Practice
GFR	Glomerular filtration rate
GH	General health
HCQ	Hydroxychloroquine
HT	Health transition
IL-1	Interleukin-1
IL-2	Interleukin-2
IL-6	Interleukin-6
IgG	Immunoglobulin G
ICH-GCP	International Conference on Harmonization-Good Clinical Practice
LLDAS	Lupus low disease activity state
LN	Lupus nephritis
MCS	Mental component summary
MH	Mental health
MHC	Major histocompatibility complex
MMF	Mycophenolate mofetil
MPA	mycophenolic acid
PCS	Physical component summary
PF	Physical functioning
PGA	Physician's Global Assessment
SAE	Serious Adverse Event
SD	Standard deviations
SDI	The Systemic Lupus International Collaborating Clinics (SLICC)/American College of Rheumatology (ACR) Damage Index
SELENA-SLEDAI	Safety of Estrogens in Lupus Erythematosus National Assessment-Systemic Lupus Erythematosus Disease Activity Index
SF-36	Short form-36
SF	Social functioning
SLE	Systemic lupus erythematosus
SLEDAI-2000	SLE Disease Activity Index-2000
SLICC	The Systemic Lupus International Collaborating Clinics
RE	Role emotional
RP	Role physical

RR	Relative risk
TNF-α	Tumor necrosis factor-α
UPCR	Urine protein-to-creatine ratio
VT	Vitality
WBC	White blood cell

2. Introduction and background

Systemic lupus erythematosus (SLE) is a disease characterized by the elevation of producing large amounts of autoantigens and then causing the production of autoantibodies, of which anti-dsDNA antibody was important. Anti-dsDNA antibody could cause organ involvement including kidney, joint, skin and so on.[1] Especially, it contributed to the pathogenesis of lupus nephritis (LN) through binding to antigens in renal cells or extracellular matrix, then triggering inflammatory activation and fibrotic processes.[2] In addition, it has been reported that renal involvement was more frequent in patients with anti-dsDNA (+) (persistent positivity) compared with anti-dsDNA (±) (initial positivity and subsequent negativity during disease course) or anti-dsDNA antibody(-) (persistent negativity).[3]

It is considered that there are three types of SLE disease activity: relapsing-remitting, chronic activity, and long quiescence,[4] with relapsing-remitting being the most common one. It has been reported that the level of anti-dsDNA antibody fluctuated with disease activity in SLE patients.[5] Flares were preceded by the increase of anti-dsDNA antibody. In other words, an elevation of anti-dsDNA antibody could occur before SLE flares.[6] A recent study reported that the prevalence of disease flares ranged from 13.0% to 15.7%.[7] Therefore, it is urgently needed to investigate the early prevention of flares in SLE patients.

Treatment of SLE consists of the use of hydroxychloroquine (HCQ), prednisone, and disease-modifying antirheumatic drugs (DMARDs), among which mycophenolate mofetil (MMF) is an important one. MMF is the pro-drug of mycophenolic acid (MPA), which can inhibit inosine monophosphate dehydrogenase that is responsible for de novo synthesis of guanosine nucleotides.[8] Besides, MPA can also regulate dendritic cell subsets to interrupt the harmful cascade of autoimmune disorders.[9] Up to now, MMF is widely used as an inducement and maintenance treatment in LN.[10,11]

It has also been reported that MMF could treat SLE patients with extra-renal involvement.[12] Therefore, in this study, we aimed to explore the efficacy and safety of MMF on new-onset SLE patients with high titers of anti-dsDNA antibody and without major organ involvement and to observe its long-term effect.

Reference:

1. Andrejevic S, Jeremic I, Sefik-Bukilica M, Nikolic M, Stojimirovic B, Bonaci Nikolic B. Immunoserological parameters in SLE: high-avidity anti-dsDNA detected by ELISA are the most closely associated with the disease activity. Clin Rheumatol. 2013;32(11):1619-1626. doi: 10.1007/s10067-013-2330-3

2. Yung S, Chan TM. Mechanisms of Kidney Injury in Lupus Nephritis - the Role of Anti-dsDNA Antibodies. Front Immunol. 2015;6:475. doi: 10.3389/fimmu.2015.00475

3. Conti F, Ceccarelli F, Perricone C, et al. Systemic Lupus Erythematosus with and without Anti-dsDNA Antibodies: Analysis from a Large Monocentric Cohort. Mediators Inflamm. 2015;2015:328078. doi: 10.1155/2015/328078

4. Petri M, Buyon J, Kim M. Classification and definition of major flares in SLE clinical trials. Lupus. 1999;8(8):685-691. doi: 10.1191/096120399680411281

5. Wang X, Xia Y. Anti-double Stranded DNA Antibodies: Origin, Pathogenicity, and Targeted Therapies. Front Immunol. 2019;10:1667. doi: 10.3389/fimmu.2019.01667

6. Ho A, Magder LS, Barr SG, Petri M. Decreases in anti-double-stranded DNA levels are associated with concurrent flares in patients with systemic lupus erythematosus. Arthritis Rheum. 2001;44(10):2342-2349. doi: 10.1002/1529-0131(200110)44:10<2342::aid-art397>3.0.co;2-8

7. Peng L, Wang Z, Li M, et al. Flares in Chinese systemic lupus erythematosus patients: a 6-year follow-up study. Clin Rheumatol. 2017;36(12):2727-2732. doi: 10.1007/s10067-017-3842-z

8. Kaltenborn A, Schrem H. Mycophenolate mofetil in liver transplantation: a review. Ann Transplant. 2013;18:685-696. doi: 10.12659/AOT.889299

9. Shigesaka M, Ito T, Inaba M, et al. Mycophenolic acid, the active form of mycophenolate mofetil, interferes with IRF7 nuclear translocation and type I IFN

production by plasmacytoid dendritic cells. Arthritis Res Ther. 2020;22(1):264. doi: 10.1186/s13075-020-02356-z
10. Ginzler EM, Dooley MA, Aranow C, et al. Mycophenolate mofetil or intravenous cyclophosphamide for lupus nephritis. N Engl J Med. 2005;353(21):2219-2228. doi: 10.1056/NEJMoa043731
11. Palmer SC, Tunnicliffe DJ, Singh-Grewal D, et al. Induction and Maintenance Immunosuppression Treatment of Proliferative Lupus Nephritis: A Network Meta-analysis of Randomized Trials. Am J Kidney Dis. 2017;70(3):324-336. doi: 10.1053/j.ajkd.2016.12.008
12. Yahya F, Jasmin R, Ng CT, Cheah TE, Sockalingam S. Open label randomized controlled trial assessing the efficacy of mycophenolate sodium against other conventional immunosuppressive agents in active systemic lupus erythematosus patients without renal involvement. Int J Rheum Dis. 2013;16(6):724-730. doi: 10.1053/j.ajkd.2016.12.008

3. Study outcomes

3.1 Primary Endpoint:

The primary outcome was the proportion of patients who have flares (mild to moderate flares and severe flares) defined by modified SELENA-SLEDAI Flare Index (SFI) [1,2] at 96 weeks of follow-up.

The definition of mild to moderate flare is the fulfillment of one or more following items:

1) variation of SELENA-SLEDAI score $\geqslant$3 (but <12);

2) new or worsening rashes, cutaneous vasculitis, nasopharyngeal ulcers, serositis, arthritis or fever caused by lupus;

3) increase in the dose of prednisone, but less than 0.5mg/kg/day;

4) increase of nonsteroidal anti-inflammatory drugs or HCQ;

5) Physician's Global Assessment (PGA) (0-3 scale) $\geqslant$1.

Severe flare is defined as the fulfillment of one or more of the following items:

1) change in SELENA-SLEDAI score of more than 12;

2) new or worsening central nervous system involvement, vasculitis, nephritis, myositis, thrombocytopenia (platelet <60*10^9/L) or haemolytic anaemia (haemoglobin <70g/L or decrease in haemoglobin level >30g/ L);

3) increased usage of prednisone (doubled in dosage or more than 0.5mg/kg/day);

4) added cyclophosphamide, azathioprine, methotrexate, mycophenolate mofetil or hospitalization because of lupus activity;

5) PGA >2.5.

3.2 Secondary Endpoint:

1) The proportion of lupus low disease activity state (LLDAS) at week 96.

2) Short form-36 (SF-36) score of before and after treatment in two groups.

3) The proportion of adverse events of two groups in the follow-ups.

4) Changes in SLEDAI-2000 score.

5) Changes in prednisone dose.

Reference:

1. Petri M, Buyon J, Kim M. Classification and definition of major flares in SLE clinical trials. *Lupus* 1999;8:685-91. doi: 10.1191/096120399680411281

2. Petri M, Kim MY, Kalunian KC, et al. Combined oral contraceptives in women with systemic lupus erythematosus. *N Engl J Med* 2005;353:2550-8. doi: 10.1056/NEJMoa051135

4. Study design

In this study, we randomly assigned patients (1:1) to Control group and MMF group. Control group received treatment with HCQ (5mg/kg/day) and prednisone (0.5mg/kg/day); while MMF group received treatment with HCQ (5mg/kg/day) and prednisone (0.5mg/kg/day) plus MMF 500mg twice daily. The dose of MMF stayed the same unless a contrary or severe adverse event occurred.

Prednisone dose was tapered as follows:

Initial dose (0.5mg/kg/day) maintained for four weeks

↓

tapered 5mg every two weeks till to 20 mg/day

↓

tapered 5mg every month

↓

gradually to 0.1-0.2mg/kg/day

The taper of prednisone doses was determined by the physicians according to the assessment of patients.

Notes:

1) Patients with severe flare were not involved in subsequent treatment plan, but they were still being followed up consistently until the end of the entire process.
2) Patients who met the criteria of severe flare were treated with increased doses of prednisone (more than 0.5mg/kg/day), immunosuppressive therapy or hospitalized for treatment according to the patients' condition and the physician's assessment.
3) Patients with mild to moderate flares continued to be followed up till the end of Week 96, and all flare episodes were recorded.
4) Patients with mild to moderate flares could increase the dose of prednisone (less than 0.5mg/kg/day) or nonsteroidal anti-inflammatory drugs.
5) For fever, rash, and arthritis, the dose of prednisone could be used at an initial dose of less than 0.5mg/kg/day, and following the assessment of symptoms, the dose could be increased to more than 0.5mg/kg/day if needed.
6) Any sign of kidney involvement (glomerular haematuria and/or cellular casts, proteinuria >0.5g/24hours (or spot urine protein-to-creatine ratio (UPCR) >500mg/g), unexplained decrease in glomerular filtration rate (GFR)), renal biopsy was suggested to confirm the diagnose of LN. Results of renal biopsy were confirmed by a senior nephrologist (Jing Xu). The treatment of LN was provided according to the European Renal Association-European Dialysis and Transplant Association (ERA-EDTA) guideline.
7) The treatment of other organ involvement was provided following the 2019 European League Against Rheumatism (EULAR) recommendation.

5. Participant selection

During enrolment, patients were required:

5.1 Inclusion Criteria:

5) Age between 18 and 65 years old;

6) Diagnosed with SLE at the time of screening by fulfilling ACR 2019 classified criteria or its 2017 version;

3) Have not received any prior SLE treatment;

4) Have a positive antinuclear antibody (HEp-2 titer ⩾1:80);

5) Have a positive anti-dsDNA antibody (fulfilled both anti-dsDNA (ELISA) ⩾ 300 IU/mL and anti-dsDNA (CLFT) ⩾1:10);

6) Do not have major organ involvement (i.e., brain, heart, liver, kidney, lung, muscle, serous cavity and gastrointestinal tract). Participants were permitted to have rash, arthritis, alopecia, oral ulcer, and mild hematologic system involvement (white blood cell >1.5*10^9/L and <4*10^9/L, hemaglobin>90g/L and <120g/L, platelet>60*10^9/L and <100*10^9/L).

5.2 Exclusion Criteria:

1) SLE patients who had been treated;

2) Liver and kidney dysfunction (Alanine aminotransferase (ALT)/ aspartate aminotransferase (AST) > 2 times upper normal limits; creatinine clearance rate < 60ml/min);

3) Cancer;

4) Recent infection or hematologic diseases not caused by SLE;

5) Pregnancy or planned to be pregnant;

6) Patients not willing to join the study.

6. Sample size calculation

We referred to the flare rate of the placebo arm in ‘Met Lupus’ trial[1] and the flare rate from the BLISS-52, BLISS-76 and BLISS-North East Asia phase III trials[2,3]. We supposed the flare rate to be 10% in MMF group and 30% in Control group. Z test with unpooled variance (alpha=0.0568, beta=0.1964, power=0.8036) was used for

sample size calculation. A sample size of 58 patients per group would provide the trial with 80% power at a two-sided alpha error of 0.05 to detect a difference between the two groups using the software PASS version 11.0. Taking an estimated 10% drop-out into account, a sample size of 65 patients per group could fulfill the statistical requirement. The sample size was calculated using PASS version 11.0 software as follows:

Two Independent Proportions (Null Case) Power Analysis

Numeric Results of Tests Based on the Difference: P1 - P2

H0: P1-P2=0. H1: P1-P2=D1<>0. Test Statistic: Z test with unpooled variance

Power	Sample Size Grp 1 N1	Sample Size Grp 2 N2	Prop|H1 Grp 1 or Trtmnt P1	Prop Grp 2 or Control P2	Diff if H0 D0	Diff if H1 D1	Target Alpha	Actual Alpha	Beta
0.8036	58	58	0.1000	0.3000	0.0000	-0.2000	0.0500	0.0568	0.1964

Reference:

1. Sun FF, Wang HJ, Liu Z, et al. Safety and efficacy of metformin in systemic lupus erythematosus: a multicentre, randomised, double-blind, placebo-controlled trial. *Lancet Rheumatol*. 2020;2(4):E210-E216.

2. Furie R, Petri M, Zamani O, et al. A phase III, randomized, placebo-controlled study of belimumab, a monoclonal antibody that inhibits B lymphocyte stimulator, in patients with systemic lupus erythematosus. *Arthritis Rheum.* 2011;63(12):3918-3930. doi: 10.1002/art.30613

3. Navarra SV, Guzman RM, Gallacher AE, et al. Efficacy and safety of belimumab in patients with active systemic lupus erythematosus: a randomised, placebo-controlled, phase 3 trial. *Lancet.* 2011;377(9767):721-731. doi: 10.1016/S0140-6736(10)61354-2

7. Analysis population

For the purposes of analysis, the following analysis sets are defined:

Participant Analysis Set	Description
Intention-To-Treat (ITT) Set	All randomized participants who received at least one dose of the intervention treatment. Participants

	will be analyzed according to the intervention to which they were allocated.
Per-protcol (PP) Set	A subset of ITT set without important protocol deviations affecting primary efficacy analysis.

8. Statistical procedures

8.1 Randomization and masking

Participants were randomly assigned (1:1) using blocks of four to receive either MMF (MMF group) or a control treatment (Control group). A statistician from the Clinical Trial Center of Ruijin Hospital, Li Jian, who was masked to trial allocation generated the randomization sequences by a computer algorithm. Then, numbers were written on the cards and packed in sequentially numbered envelopes that were opened when the patients were enrolled. Dr. Junna Ye assigned patients to different groups according to the number on the card. Assessors were masked to the group allocation. Eligible subjects were recruited from the Department of Rheumatology and Immunology of Ruijin Hospital and the Department of Rheumatology and Immunology of the other two clinical centers. Doctors were responsible for patient inclusion and regular follow-up assessments (Dr. Honglei Liu, Xiaobing Cheng, Yutong Su, Xiaowei Chen, and Hui Zheng). The assessment of patient flare was strictly based on the SELENA-SLEDAI Flare Index (including severe flare, and mild to moderate flare) rather than subjective decisions. Data analysts (Yijun You and Zhuochao Zhou) were not involved in patient assessment and treatment, and they were masked to the group allocation.

8.2 Analysis principles

1) For the primary outcome assessed at two levels (mild-to-moderate and severe flares), Bonferroni correction was used and two-sided at a significance level of 0.025. Other tests were performed two-sided at the significance level of 0.05. The confidence level for all confidence intervals (CI) will be 95%.

2) The number of observations used in the analysis will be reported.

3) The statistical analysis plan is drafted prior to database lock, and it includes a detailed description of the statistical analyses. The statistical analyses were performed using SPSS Statistics (version 23.0), GraphPad Prism (version 8.0), and R software

(version 4.0.0). Figures will be plotted using GraphPad Prism (version 8.0) and R software (version 4.0.0).

8.3 Data quality control

All responsible data collectors will be trained at the beginning of this study (GCP training). Double entry is required for all variables and double validation is required for all other variables. The hospital coordinator at each collaborating site ensures that all data needed are collected.

8.4 Patients characteristics and baseline comparisons

Description and statistical inference of the following baseline characteristics will be presented for Control group and MMF group.

In the descriptive statistics, data were expressed as frequencies (percentages) for categorical variables and medians (interquartile) or means ± standard deviations (SD) for continuous variables. Additionally, t-tests were used to compare between-group differences in continuous variables after exploring the normality of data distribution using the Shapiro-Wilk test. Mann-Whitney U tests were used to compare continuous variables with skewed distributions. Pearson Chi-square or Fisher's exact tests were used to compare between-group differences in categorical variables. Relative risk (RR) was used to compare the proportion of patients with the endpoint between the MMF and Control groups. We estimated the severe flare-free survival proportion of the MMF group and Control group using the Kaplan-Meier method with a log rank test. Hazard ratios (HR) were calculated by Cox proportional-hazards model.

8.5 Primary outcome

Flare proportion was defined as categorical variables, data were expressed in the form of frequency (percentages). The primary outcome was the proportion of patients with flares (including mild-to-moderate flares and severe flares) during follow-up. Safety of Estrogens in Lupus Erythematosus National Assessment-Systemic Lupus Erythematosus Disease Activity Index (SELENA-SLEDAI) Flare Index (SFI) was used for assessing disease flares in this trial. Pearson Chi-square or Fisher's exact tests were used to compare between-group differences. We first calculated the total flare rate, and then calculated mild-to-moderate flare rate and severe flare rate, respectively.

For the primary outcome, Bonferroni correction was used and two-sided at a significance level of 0.025. A more stringent statistical criterion was used. Relative risk (RR) was employed to compare the proportion of patients with the end point between MMF group and Control group. Severe flare-free survival proportion was estimated by using the Kaplan-Meier method in the intention to MMF group and Control group with a log rank test. Regarding patients who dropped out of the study, we collected all of the data during follow-up period. For patient who loss to follow-up, we did not collect the data for the subsequent time after lost to follow-up event. We used the ITT set for primary outcome calculation. All of the patients were included in the denominator. We estimated the severe flare-free survival proportion of the MMF group and Control group in both ITT and PP Set using the Kaplan-Meier method.

8.6 Secondary outcomes

Secondary Endpoint including:

1) The proportion of lupus low disease activity state (LLDAS) at week 96.

2) Short form-36 (SF-36) score of before and after treatment in two groups.

3) The proportion of adverse events of two groups in the follow-ups.

4) Changes in SLEDAI-2000 score.

5) Changes in prednisone dose.

In descriptive statistics, data were expressed in the form of frequency (percentages) for categorical variables (proportion of LLDAS, proportion of adverse events (AEs)), medians (interquartile) or means ± standard deviations (SD) for continuous variables (SF-36 score variation, SLEDAI-2000 score variation and prednisone dose).

In addition, t-tests were used to compare between-group differences in continuous variables after exploring the normality of data distribution using the Shapiro-Wilk test. Mann-Whitney U tests were used to compare continuous variables with a skewed distribution. Pearson Chi-square or Fisher's exact tests were used to compare between-group differences in categorical variables. All tests were performed two-sided at the significance level of 0.05.

8.7 Other variables

In our trial, changes in serological markers, including anti-dsDNA antibody, immunoglobulin G (IgG), erythrocyte sedimentation rate (ESR), and C-Reactive Protein (CRP) were also assessed in the two groups.

The Systemic Lupus International Collaborating Clinics (SLICC)/American College of Rheumatology (ACR) Damage Index (SDI) was also compared in the two groups. Statistical analysis methods will be used appropriately for those variables.

9. Flow chart

Flowchart : see Figure 1.

10. Statistical software

The statistical analyses were performed using SPSS Statistics (version 23.0), GraphPad Prism (version 8.0), and R software (version 4.0.0). Figures will be plotted using GraphPad Prism (version 8.0) and R software (version 4.0.0).

11. Study centers list

Study centers include：

1) Ruijin Hospital, Shanghai Jiao Tong University School of Medicine;

2) The First Affiliated Hospital of Wenzhou Medical University, Wenzhou Medical University;

3) The Second Affiliated Hospital of Shandong First Medical University, Shandong First Medical University.

12. Study content schedule

time / content	Screening period	Follow-up period			
	Screening day	Week 24	Week 48	Week 72	Week 96
Informed consent form	√				
Inclusion/Exclusion Criterias	√				
Physical examination	√	√	√	√	√
SLEDAI-2000 Index	√	√	√	√	√
Blood routine examination	√	√	√	√	√
Urine routine examination	√	√	√	√	√

ALT&AST	√	√	√	√	√
Scr	√	√	√	√	√
ESR & CRP	√	√	√	√	√
Complement 3 & 4	√	√	√	√	√
Anti-dsDNA antibody	√	√	√	√	√
immunoglobulin G	√	√	√	√	√
SF-36 score	√	√	√	√	√
Medication	√	√	√	√	√
Adverse events	√	√	√	√	√

13. Ethics

The study was approved by the Institutional Research Ethics Committee of Ruijin Hospital (ID: 2018-120) and the local authorized ethics committee of the other two clinical centers. All patients provided written informed consent.

14. Adverse events disposition

Adverse events will be recorded during the follow-ups. Proper treatment would be given to deal with the adverse events.

15. Tables and figures

CONSORT Flow Diagram

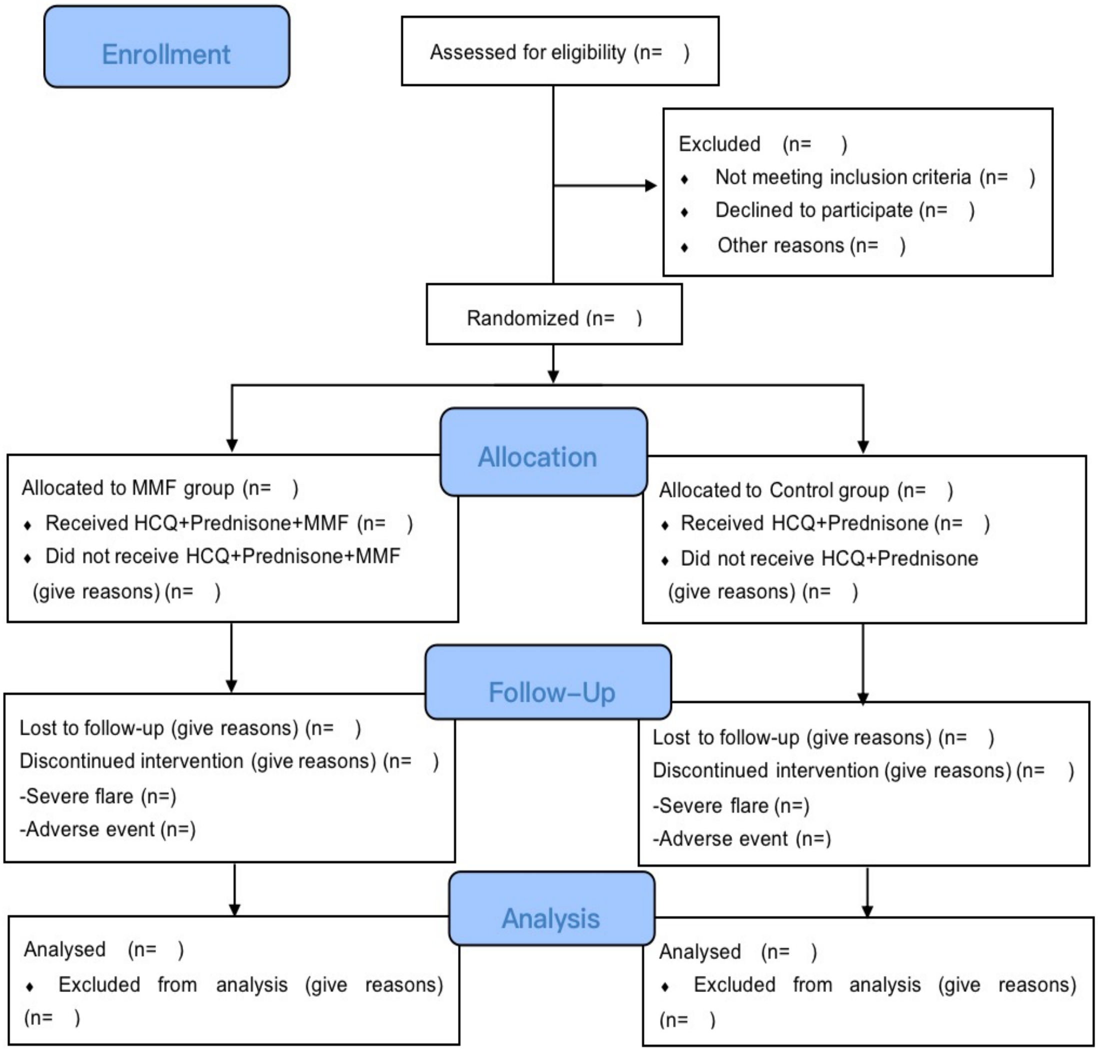

Figure 1 : Flowchart

Table 1 Baseline characteristics

	Control group (n=)	MMF group (n=)
	Median(quartile25%,75%) or N (%)	Median(quartile25%,75%) or N (%)

Age(years)
Female, N (%)
Disease duration(months)
SLEDAI-2000 score
System involvement
Arthritis, N (%)
Rash, N (%)
Fever, N (%)
Alopecia, N (%)
Oral ulcer, N (%)
Leukopenia, N (%)
Thrombocytopenia, N (%)
Laboratory results
Anti-dsDNA antibody (IU/mL)
Low C3 level, N (%)
Low C4 level, N (%)
WBC (*10^9/L)
Hemoglobin (mg/L)
Platelet (*10^9/L)
ESR (mm/h)
CRP (mg/L)

SLEDAI: Systemic lupus erythematosus disease activity index; ESR: Erythrocyte sedimentation rate; CRP: C-Reactive Protein; WBC: White blood cell; MMF: Mycophenolate mofetil

Table 2 and followings: MMF efficacy versus Control at visit xx

	Control group (n=)	MMF group (n=)
SLEDAI-2000 Index, mean		
Blood routine examination, mean		
Urine routine examination, mean		
ALT&AST, mean		
Scr, mean		
ESR, mean		
CRP, mean		
Complement 3 & 4, mean		
Anti-dsDNA antibody, mean		
Immunoglobulin G, mean		

SLEDAI: Systemic lupus erythematosus disease activity index; ALT: Alanine aminotransferase; AST:Aspartate aminotransferase; Scr: Serum creatinine; ESR: Erythrocyte sedimentation rate; CRP: C-Reactive Protein; MMF: Mycophenolate mofetil

Table 3 Outcomes of 96-week follow-ups.

	Control group	MMF group	RR	*P* value

	(n=)	(n=)	
	Mean (SD) or N (%)	Mean (SD) or N (%)	(95%CI)
**Primary outcome, %**			
Proportion of severe flares			
Proportion of mild to moderate flares			
**Severe flares, %**			
Lupus nephritis			
Neuropsychiatric symptoms			
Rash (prednisone dose > 0.5mg/kg/day)			
Pleurisy			
Thrombocytopenia (<60* 10^9/L)			
Pulmonary arterial hypertension			
Hemolytic anemia			
Fever (prednisone dose > 0.5mg/kg/day)			
Henoch-Schonlein purpura			
Moderate mitral insufficiency			
Arthritis (prednisone dose > 0.5mg/kg/day)			

**Mild to moderate flares, %**
Arthritis
Rash
Oral ulcer
Fever
Leukopenia (1.5-4*10^9/L)
Thrombocytopenia (60-100*10^9/L)
**Secondary outcome, %**
Proportion of LLDAS, %
SLEDAI-2000 score at last visit
Prednisone at last visit (mg)

LLDAS: Lupus low disease activity state; SLEDAI: Systemic lupus erythematosus disease activity index;

Table 4 Adverse events.

	Control group (n=) N (%)	MMF group (n=) N (%)	*P* value
Total AEs, %			
Infection			
Upper respiratory tract			
Pneumonia			
Lower urinary tract			

Herpes zoster virus

Candida

Tuberculosis

Gastrointestinal event

Bone fracture

Osteonecrosis of the femoral head

Other events

Table 5. The comparison of SF-36 scores before and after treatment.

SF-36: Short Form-36; PF: Physical functioning; BP: Bodily pain; MH: Mental

		PF	BP	MH	RP	VT	SF	GH	RE	HT	MCS	PCS
Control group	Before treatment											
	After treatment											
	Change in score											
MMF group	Before treatment											
	After treatment											
	Change in score											

health; RP: Role physical; VT: Vitality; SF: Social functioning; GH: General health; RE: Role emotional; HT: Reported health transition; PCS: Physical component summary; MCS: Mental component summary.

Table 6. The comparison of organ damage according to The Systemic Lupus International Collaborating Clinics (SLICC)/American College of Rheumatology (ACR) Damage Index (SDI) for SLE.

	Control group (n=) N (%)/ mean (SD)	MMF group (n=) N (%)/mean (SD)	*p* value
SDI, %			
SDI, mean			
