# Supplementary material for: Mycophenolate Mofetil and New-Onset Systemic Lupus Erythematosus: A Randomized Clinical Trial
Source: JAMA Netw Open. 2024 Sep 16;7(9):e2432131. doi: 10.1001/jamanetworkopen.2024.32131 (PMC11406395; doi:10.1001/jamanetworkopen.2024.32131)
Supplement: Supplement 2. — eTable 1. Histopathological Details on LN Classification in Control Group and MMF Group eTable 2. Comparison of SF-36 Scores Before and After Treatment in Control Group and MMF Group eTable 3. The Comparison of Organ Damage in Control Group and MMF Group During 96-Week Follow-Up According to SDI for SLE eTable 4. The Details of Organ Damage in Control Group and MMF Group During 96-Week Follow-Up According to SDI for SLE eTable 5. SLEDAI-2000 and Prednisone Dose in Control Group and MMF Group at Week 96 eAppendix 1. List of Safety of Estrogens in Lupus Erythematosus National Assessment-Systemic Lupus Erythematosus Disease Activity Index (SELENA-SLEDAI) Flare Index (SFI) eAppendix 2. List of LLDAS Definition eFigure 1. Clinical Features of the Control and MMF Groups eFigure 2. Pretreatment and Posttreatment SF-36 Scores in Control Group and MMF Group [file jamanetwopen-e2432131-s002.pdf]

Supplementary Online Content

You Y, Zhou Z, Wang F, et al. Mycophenolate mofetil and new-onset systemic lupus erythematosus: a randomized clinical trial. *JAMA Netw Open*. 2024;7(9):e2432131. doi:10.1001/jamanetworkopen.2024.32131

eTable 1. Histopathological Details on LN Classification in Control Group and MMF Group

eTable 2. Comparison of SF-36 Scores Before and After Treatment in Control Group and MMF Group

eTable 3. The Comparison of Organ Damage in Control Group and MMF Group During 96-Week Follow-Up According to SDI for SLE

eTable 4. The Details of Organ Damage in Control Group and MMF Group During 96-Week Follow-Up According to SDI for SLE

eTable 5. SLEDAI-2000 and Prednisone Dose in Control Group and MMF Group at Week 96

eAppendix 1. List of Safety of Estrogens in Lupus Erythematosus National Assessment-Systemic Lupus Erythematosus Disease Activity Index (SELENA-SLEDAI) Flare Index (SFI)

eAppendix 2. List of LLDAS Definition

eFigure 1. Clinical Features of the Control and MMF Groups

eFigure 2. Pretreatment and Posttreatment SF-36 Scores in Control Group and MMF Group

This supplementary material has been provided by the authors to give readers additional information about their work.

© 2024 You Y et al. *JAMA Network Open*.

eTable 1. Histopathological Details on LN Classification in Control Group and MMF Group

	Control group	MMF group
Patient 1	Type III, activity score 4 points, chronicity index 2 points	
Patient 2	Type IV, activity score 12 points, chronicity index 4 points	
Patient 3	Type V+III, activity score 7 points, chronicity index 2 points	
Patient 4	Type III with tubulointerstitial nephritis, activity score 7 points, chronicity index 2 points	
Patient 5	Type IV, activity score 14 points, chronicity index 4 points	
Patient 6	Type III, activity score 11 points, chronicity index 4 points	
Patient 7	Type IV+V, activity score 9 points, chronicity index 3 points	
Patient 8	Type IV+V, activity score 21 points, chronicity index 3 points	
Patient 9		Type IV, activity score 8 points, chronicity index 2 points

© 2024 You Y et al. *JAMA Network Open*.

eTable 2. Comparison of SF-36 Scores Before and After Treatment in Control Group and MMF Group

		PF	BP	MH	RP	VT	SF	GH	RE	HT	MCS	PCS
Control group	Before treatment	83.2	79.9	62.8	62.9	59.8	77.0	50.5	58.1	40.6	64.2	69.9
	After treatment	88.6	84.2	67.5	77.6	69.5	80.5	60.6	74.0	64.8	72.9	77.8
	Change in score	5.4	4.3	4.8	14.7	9.7	3.5	10.1	15.9	24.2	8.6	7.9
MMF group	Before treatment	79.6	67.6	66.7	52.6	61.6	70.8	54.3	67.2	42.2	66.5	63.9
	After treatment	86.9	83.4	70.3	75.9	69.1	85.2	63.9	84.0	64.7	76.3	77.1
	Change in score	7.3	15.9	3.6	23.3	7.6	14.4	9.6	16.7	22.5	9.8	13.2
Within Control group（*p* value）		0.08	0.34	0.09	0.05	0.02	0.46	0.02	0.03	0.003	0.02	0.03
Within MMF group（*p* value）		0.13	0.01	0.43	0.03	0.06	0.01	0.01	0.13	0.02	0.02	0.01
Control group compared to MMF group（*p* value）		0.84	0.85	0.87	0.82	0.70	0.16	0.31	0.55	0.85	0.69	0.79

SF-36: Short Form-36; PF: Physical functioning; BP: Bodily pain; MH: Mental health; RP: Role physical; VT: Vitality; SF: Social functioning; GH: General health; RE: Role emotional; HT: Reported health transition; PCS: Physical component summary; MCS: Mental component summary.

© 2024 You Y et al. *JAMA Network Open*.

eTable 3. The Comparison of Organ Damage in Control Group and MMF Group During 96-Week Follow-Up According to SDI for SLE

SDI	Control group (n=65) N (%)/ mean (SD)	MMF group (n=65) N (%)/mean (SD)	*p* value
N (%)	5 (7.7%)	2 (3.1%)	0.2
mean (SD)	0.1 (0.3)	0.1 (0.3)	0.4

SDI: The Systemic Lupus International Collaborating Clinics (SLICC)/American College of Rheumatology (ACR) Damage Index

eTable 4. The Details of Organ Damage in Control Group and MMF Group During 96-Week Follow-Up According to SDI for SLE

	Control group	MMF group
Patient 1	Pulmonary arterial hypertension	
Patient 2	Steroid-related diabetes mellitus	
Patient 3	Breast cancer	
Patient 4	Moderate mitral insufficiency	
Patient 5	Phlebothrombosis	
Patient 6		Osteonecrosis of the femoral head (double hips)
Patient 7		Cervical cancer

SDI: Systemic Lupus International Collaborating Clinics (SLICC)/American College of Rheumatology (ACR) Damage Index

© 2024 You Y et al. *JAMA Network Open*.

eTable 5. SLEDAI-2000 and Prednisone Dose in Control Group and MMF Group at Week 96

	Control group (n=65)	MMF group (n=65)	*p* value
SLEDAI-2000 score at last visit, median (IQR)	2 (0-4)	2 (0-4)	0.9
Prednisone at last visit, median (IQR) , mg	5 (2.5-7.5)	3.1 (2.5-7.5)	0.6

© 2024 You Y et al. *JAMA Network Open*.

eAppendix 1. List of Safety of Estrogens in Lupus Erythematosus National Assessment-Systemic Lupus Erythematosus Disease Activity Index (SELENA-SLEDAI) Flare Index (SFI)

The definition of mild to moderate flare is the fulfillment of one or more following items: (a) variation of SELENA-SLEDAI score ≥3 (but <12);

(b) new or worsening rashes, cutaneous vasculitis, nasopharyngeal ulcers, serositis, arthritis or fever caused by lupus;

(c) increase in the dose of prednisone, but less than 0.5mg/kg/day;

(d) increase of nonsteroidal anti-inflammatory drugs or HCQ;

(e) Physician's Global Assessment (PGA) (0-3 scale) ≥1 but ≤ 2.5.

Severe flare is defined as the fulfillment of one or more of the following items:

(a) change in SELENA-SLEDAI score of more than 12;

(b) new or worsening central nervous system involvement, vasculitis, nephritis, myositis, thrombocytopenia (platelet <60*10^9/L) or haemolytic anaemia (haemoglobin <70g/L or decrease in haemoglobin level >30g/ L);

(c) increased usage of prednisone (doubled in dosage or more than 0.5mg/kg/day);

(d) added cyclophosphamide, azathioprine, methotrexate, mycophenolate mofetil or hospitalization because of lupus activity;

(e) PGA >2.5.

© 2024 You Y et al. *JAMA Network Open*.

eAppendix 2. List of LLDAS Definition

(a) SLE Disease Activity Index (SLEDAI)-2000 ≤4, with no activity in major organ systems (kidney, central nervous system (CNS), cardiopulmonary, vasculitis, fever) and no hemolytic anemia or gastrointestinal activity;

(b) no new lupus disease activity compared with the previous assessment;

(c) a Safety of Estrogens in Lupus Erythematosus National Assessment (SELENA)-SLEDAI physician global assessment (scale 0– 3) ≤1;

(d) a current prednisolone (or equivalent) dose ≤7.5 mg daily;

(e) well tolerated standard maintenance doses of immunosuppressive drugs and approved biological agents.

© 2024 You Y et al. *JAMA Network Open*.

eFigure 1. Clinical Features of the Control and MMF Groups

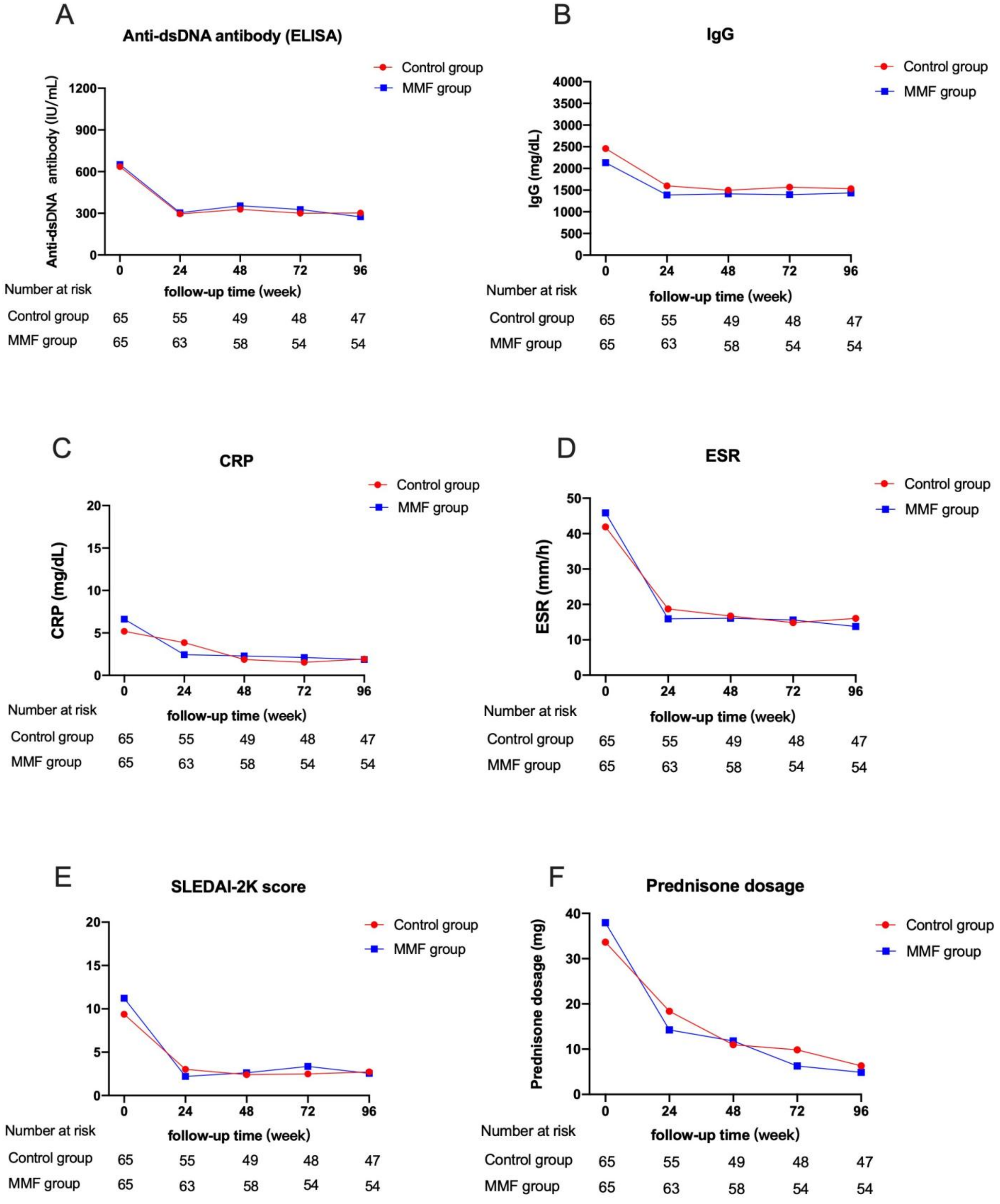

© 2024 You Y et al. *JAMA Network Open*.

(A) Anti-dsDNA antibody levels in the Control and MMF groups during follow-up. **(B)** IgG levels in the Control and MMF groups during follow-up. **(C)** CRP levels in the Control group and MMF group during follow-up. **(D)** ESR levels in the Control group and MMF group during follow-up. **(E)** SLEDAI-2000 score variation in the Control group and MMF group during follow-up. **(F)** Prednisone dosage variation in the Control group and MMF group during follow-up (Red line represented Control group and blue line represented MMF group). Note: IgG: Immunoglobulin G; SLEDAI: Systemic lupus erythematosus disease activity index; ESR: Erythrocyte sedimentation rate; CRP: C-Reactive Protein

© 2024 You Y et al. *JAMA Network Open*.

eFigure 2. Pretreatment and Posttreatment SF-36 Scores in Control Group and MMF Group

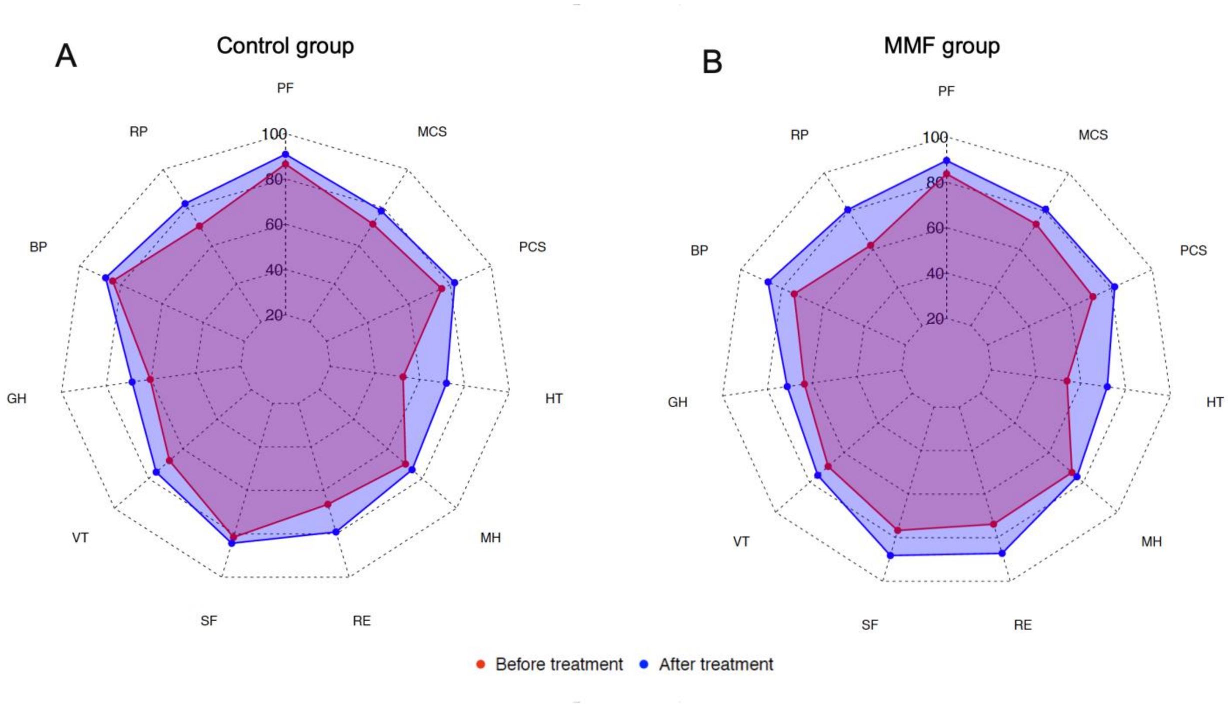

The red color represents pre-treatment and the blue color represents post-treatment.

PF: Physical functioning; BP: Bodily pain; MH: Mental health; RP: Role physical; VT: Vitality; SF: Social functioning; GH: General health; RE: Role emotional; HT: Reported health transition; PCS: Physical component summary; MCS: Mental component summary.

© 2024 You Y et al. *JAMA Network Open*.
