# Supplementary material for: Mycophenolate Mofetil and New-Onset Systemic Lupus Erythematosus: A Randomized Clinical Trial
Source: JAMA Netw Open. 2024 Sep 16;7(9):e2432131. doi: 10.1001/jamanetworkopen.2024.32131 (PMC11406395; doi:10.1001/jamanetworkopen.2024.32131)
Supplement: Supplement 3. — Data Sharing Statement [file jamanetwopen-e2432131-s003.pdf]

Data Sharing Statement

You. Mycophenolate Mofetil and New-Onset Systemic Lupus Erythematosus. *JAMA Netw Open*. Published September 09, 2024. doi:10.1001/jamanetworkopen.2024.32131

Data
Data available: Yes
Data types: Deidentified participant data
How to access data: All requests should be addressed to the corresponding author Junna Ye (E-mail address: yjn0912@qq.com)
When available: With publication

Supporting Documents
Document types: Other (please specify)
Additional Information: Trial approved study protocol, Statistical analytical plan
How to access documents: We will be happy to share with the journal and post online upon publication
When available: With publication

Additional Information
Who can access the data: researchers whose proposed use of the data has been approved
Types of analyses: For research only
Mechanisms of data availability: with a signed data access agreement
